# Synthesis, DFT Molecular Geometry and Anticancer Activity of Symmetrical 2,2′-(2-Oxo-1*H*-benzo[*d*]imidazole-1,3(2*H*)-diyl) Diacetate and Its Arylideneacetohydrazide Derivatives

**DOI:** 10.3390/ma15072544

**Published:** 2022-03-30

**Authors:** Manel Dhahri, Firdos Alam Khan, Abdul-Hamid Emwas, Rua B. Alnoman, Mariusz Jaremko, Nadjet Rezki, Mohamed Reda Aouad, Mohamed Hagar

**Affiliations:** 1 Biology Department, Faculty of Science Yanbu, Taibah University, Yanbu El-Bahr 46423, Saudi Arabia; manel.dhari@gmail.com; 2 Department of Stem Cell Research, Institute for Research and Medical Consultations (IRMC), Imam Abdulrahman Bin Faisal University, Dammam 31441, Saudi Arabia; fakhan@iau.edu.sa; 3 Core Labs, King Abdullah University of Science and Technology, Thuwal 23955-6900, Saudi Arabia; abdulhamid.emwas@gmail.com; 4 Chemistry Department, Faculty of Science, Taibah University, Yanbu Branch, Yanbu 46423, Saudi Arabia; rua-b-n@live.co.uk; 5 Smart-Health Initiative (SHI) and Red Sea Research Center (RSRC), Division of Biological and Environmental Sciences and Engineering (BESE), King Abdullah University of Science and Technology (KAUST), Thuwal 23955-6900, Saudi Arabia; mariusz.jaremko@kaust.edu.sa; 6 Department of Chemistry, College of Science, Taibah University, Al-Madinah Al-Munawarah 30002, Saudi Arabia; nadjetrezki@yahoo.fr (N.R.); aouadmohamedreda@yahoo.fr (M.R.A.); 7 Chemistry Department, Faculty of Science, Alexandria University, Alexandria 21321, Egypt

**Keywords:** ultrasonic synthesis, benzimidazole-acetohydrazide, configurational-conformational-DFT study, anticancer agent, colon cancer, cervical cancer, cell viability

## Abstract

To identify new candidate anticancer compounds, we here report the synthesis of benzimidazole derivatives: diethyl 2,2′-(2-oxo-1*H*-benzo[*d*]imidazole-1,3(2*H*)-diyl) diacetate and its arylideneacetohydrazide derivatives, using ultrasonic irradiation and conventional heating. The compounds were confirmed by Nuclear magnetic resonance (NMR) (JEOL, Tokyo, Japan) and Fourier transform infrared spectroscopy (FTIR) spectroscopy (Thermoscientific, Waltham, MA, USA). The molecular structure and electronic properties of the studied compounds were predicted for the acetohydrazide hydrazones. These compounds exist as a mixture of configurational and conformational isomerism as well as amido-amidic acid tautomerism. The NMR spectral data proved the predominance of *syn-E* amido isomers. In addition, density functional theory (DFT) predicted stability in the gas phase and showed that *syn-E* amido isomers are the most stable in the presence of an electron donating group, while the anti-isomer is the most stable in the presence of electron-attracting substituents. The anticancer activity of these synthetic compounds **6a**, **6b** and **6c** towards both colon cancer (HCT-116) and cervical cancer (HeLa) cells was examined by MTT assay and DAPI staining. The MTT assay revealed a strong antiproliferative effect against the cancer cells at low concentrations, and interestingly, no significant inhibitory action against the non-cancerous cell line, HEK-293. The IC50 values for HCT-116 were 29.5 + 4.53 µM, 57.9 + 7.01 µM and 40.6 + 5.42 µM for **6a**, **6b**, and **6c**, respectively. The IC50 values for HeLa cells were 57.1 + 6.7 µM, 65.6 + 6.63 µM and 33.8 + 3.54 µM for **6a**, **6b**, and **6c**, respectively. DAPI staining revealed that these synthesized benzimidazole derivatives caused apoptotic cell death in both the colon and cervical cancer cells. Thus, these synthetic compounds demonstrate encouraging anticancer activity as well as being safe for normal human cells, making them attractive candidates as anticancer agents.

## 1. Introduction

Cancer is one of the world’s biggest medical challenges [1]. Cancer is characterized by cell cycle disruption, which inhibits cell differentiation and promotes uncontrollable cell growth [2]. Fighting cancer necessitates the use of appropriate treatment protocols. While several treatment options exist, such as chemotherapy, immunotherapy, and radiotherapy, their use is limited due to intolerable side effects and the development of drug resistance [3]. To counter this, scientists are continuously developing new candidates for cancer treatment. For instance, the heterocyclic compounds, benzimidazole derivatives, have recently attracted a lot of attention due to their anticancer properties, and have shown to be promising candidates for the treatment of cancer [4,5].

In general, heterocycles perform an important role in both pharmaceutical and organic chemistry, and much research has been devoted to their synthesis. Nitrogen heterocycles, in particular, possess a wide range of biological and pharmacological properties [4]. Benzimidazole derivatives are widely used in the production of a variety of drugs [6,7,8]. These active heterocycles interact with a wide range of biological targets due to the benzene and imidazole components [3]. One of the valuable properties of imidazole is its strong hydrogen bonding ability and its high affinity for the metal active sites present in many proteins [9,10,11]. The benzimidazole skeleton is present in a number of pharmaceutical agents such as anticancer agents, antihypertensives, antifungals, antiulceratives, antivirals, and antihistamines [12,13,14]. 

Studying the tautomerism of organic compounds is important because different tautomers differently utilize the metabolic products and related compounds involved in enzymatic biological reactions [15,16,17,18,19,20]. Moreover, we have a strong interest in using theoretical calculations to predict molecular geometry and to investigate their effects on the compound’s properties and incorporating them with experimentally determined properties [21,22,23,24].

Nowadays, it is also important to be aware of the need to protect natural resources by developing environmentally friendly processes and lowering energy consumption [25,26,27,28]. Ultrasound irradiation has been an effective tool in organic synthesis over the last twenty years [29,30,31,32,33,34,35]. It is a valuable tool used to accelerate reactions and is important in green chemistry for minimizing waste and energy requirements [36]. The usage of ultrasound in chemical reactions in solution offers specific activation attributed to a physical phenomenon: acoustic cavitation. Cavitation is a process in which mechanical activation destroys the attractive forces of molecules in the liquid phase [37,38].

The aim of this work was to study the effectiveness of ultrasound irradiation for the synthesis of new potential anticancer candidates, 2,2′-(2-oxo-1*H*-benzo[*d*]imidazole-1,3(2*H*)-diyl)diacetic acid and its arylideneacetohydrazide derivatives, as well as to perform DFT/B3LYP calculations to study the molecular structure and electronic characteristics. In addition, their anticancer activity was investigated using human colon cancer and cervical cancer cells by the 3-(4,5-dimethylthiazol-2-yl)-2,5-diphenyl-2H-tetrazolium bromide (MTT) assay and the 4′,6-diamidino-2-phenylindole (DAPI) staining [39,40,41]. 

## 2. Materials and Methods

### 2.1. General

IR spectra were measured by a Nicolet iS 10 Thermoscientific IR spectrophotometer (Thermoscientific, Waltham, MA, USA). The ultraviolet spectra were recorded with UV–Vis spectrophotometer model UV-1800 SHIMADZU (SHIMADZU, Kyoto, Japan). NMR spectra were measured with a JEOL spectrometer (JEOL, Tokyo, Japan) using tetramethylsilane as a reference. Thin layer chromatography (TLC) were performed on Merck Kiesel gel; fluorescent plates.

### 2.2. Preparation of 1H-Benzo[a]imidazol-2(3H)-2-one (***2***)

A mixture of 1,2-phenylenediamine (0.5 g, 4.6 mmol) and urea (0.26 g, 9.2 mmol) in Dimethyl formamide (DMF)(5 mL) was subjected to ultrasonic irradiation for 40 min. The solvent was concentrated under reduced pressure, poured into water, and the formed precipitate was filtered and washed with dichloromethane to afford the titled compound as a pale yellow solid; m.p. 100–102 °C [42]; Yield: 90%; IR: 3150 (NH), 3049 (CH ar), 1690 (CO), 1619 (C=N), 1429 (C=C), 1373.74, 1197.32, 1164.03, 749.65, 701.67 cm^−1^ [43].

### 2.3. General Method for Alkylation Reaction

A mixture of 1*H*-benzo[*d*]imidazol-2(3*H*)-one (134 mg, 0.01 mol), potassium carbonate or sodium carbonate (0.03 mol) and ethyl chloroacetate (1.33 mL, 0.04 mol) in 5 mL of DMF or acetone was subjected to ultrasound for 30 min. The reaction mixture was concentrated under reduced pressure, then 100 g of ice water was added, then neutralized with 1 M HCl. The formed precipitate was collected by filtration, washed with ethanol then with water, and recrystallized from hot water. The reaction proceeded not to a dialkylated product but gave a mixture of mono and dialkylated products.

### 2.4. Preparation of Diethyl N,N′-2,2′-(2-Oxo-1H-benzo[d]imidazole-1,3(2H)-diyl)diacetate (***3***)

A mixture of 1*H*-benzo[*d*]imidazol-2(3*H*)-one (134 mg, 0.01 mol), sodium hydride (0.4 g, 0.02 mol) and ethyl chloroacetate (1.33 mL, 0.04 mol) in 5 mL of DMF was subjected to ultrasound for 15 min at room temperature. The reaction mixture was added to 100 g of ice water. The precipitate was collected by filtration, and washed with water and ethanol. The product was recrystallized from hot water. The crystals were colorless: yield = 275 mg (90%), m.p. 215 °C, ^1^H NMR (600 MHz, DMSO) δ 7.17 (dd, *J* = 5.8, 3.2 Hz, 2H), 7.09 (dd, *J* = 5.8, 3.2 Hz, 2H), 4.73 (s, 4H), 4.15 (q, *J* = 7.1 Hz, 4H), 1.20 (t, *J* = 7.1 Hz, 6H) (Figure S8). ^13^C NMR (101 MHz, DMSO) δ 166.12 (C=O), 166.45(C=O), 154.32, 130.12, 121.55, 108.34, 54.99(CH2), 42.69(CH2), 14.54(CH3).

### 2.5. Preparation of N,N′-2,2′-(2-Oxo-1H-benzo[d]imidazole-1,3(2H)-diyl)diacetic Acid (***4***)

A mixture containing 1*H*-benzo[*d*]imidazol-2(3*H*)-one (134 mg, 0.01 mol), 5 mL of DMF and) potassium carbonate (1.68 g, 0.03 mol and sodium iodide (0.5 g) were added. Ethyl chloroacetate (1.33 mL, 0.04 mol) was added in one portion, and the reaction was subjected to ultrasound for 30 min. The reaction mixture was added to 50 g of ice water then neutralized with 1 M HCl. The formed precipitate was collected by filtration, washed with water, and recrystallized from hot water. White crystals were produced: yield (1.0 g, 58%), m.p. 288 °C, ^1^HNMR (600 MHz, DMSO) δ = 9.9 (s, 1H), 7.16 (dd, *J* = 5.7, 3.2 Hz, 2H), 7.07 (dd, *J* = 5.8, 3.2 Hz, 2H), 4.62 (s, 4H) (Figure S9). ^13^C NMR (101 MHz, DMSO) δ 168.56 (C=O), 166.23(C=O), 154.67, 130.23, 121.76, 108.32, 42.66(CH2).

### 2.6. Synthesis of 2,2′-(2-Oxo-1H-benzo[d]imidazole-1,3(2H)-diyl)diacetohydrazide (***5***)

A mixture of diethyl 2,2′-(2-oxo-1*H*-benzo[*d*]imidazole-1,3(2*H*)-diyl) diacetate (306 mg, 0.01 mol), and 3 mL of hydrazine hydrate in 25 mL of ethanol was either refluxed for 2 h or subjected to ultrasound for 20 min at 60 °C. The reaction mixture was cooled in an ice bath. The precipitate was collected by filtration, and washed with ethanol. The product was recrystallized from hot ethanol. Colorless crystals were produced: yield = 250 mg (90%), m.p. >300 °C [43]. ^1^HNMR (500 MHz, DMSO) δ = 9.31 (s, 1.7H, NH), δ = 8.65 (s, 0.3H, NH), 7.04–6.98 (m, 4H, Ar-H), 4.76 (s, 0.3H, CH2), 4.41 (s, 3.7H, CH2), 4.31 4.31 (s, 4H, NH2) (Figure S10). ^13^C NMR (101 MHz, DMSO) δ 166.70 (C=O), 166.67(C=O), 154.09, 130.00, 121.57, 108.65, 42.69(CH2) (Figure S11).

### 2.7. Synthesis of N,N′-2,2′-(2-Oxo-1H-benzo[d]imidazole-1,3(2H)-diyl)bis(arylidene) Acetohydrazide (***6a***–***c***)

2,2′-(2-oxo-1*H*-benzo[*d*]imidazole-1,3(2*H*)-diyl)diacetohydrazide (278 mg, 0.01 mol), and *p*-flourobenzaldehyde, *p*-nitrobenzaldehyde or *p*-methoxybenzaldehyde (0.02 mol) in 25 mL of ethanol were subjected to ultrasound for 30 min at 60 °C. The precipitate was collected by filtration while hot, and washed with hot ethanol. The product was recrystallized from DMF.

### 2.8. N,N′-2,2′-(2-Oxo-1H-benzo[d]imidazole-1,3(2H)-diyl)bis(4-methoxybenzylidene) Acetohydrazide) (***6a***)

Colorless crystals were produced: yield = 488 mg (90%), m.p. >300 °C. ^1^H NMR (400 MHz, DMSO) δ 11.67 (s, 0.48H, NH), 11.60 (s, 1.30H, NH *syn* isomer), 8.18 (s, 0.5H, CH=N *anti* isomer), 8.00 (s, 1.40H, CH=N *syn* isomer), 7.70 (d, *J* = 8.3 Hz, 2.57H, aromatic H *syn* isomer), 7.65 (d, *J* = 8.2 Hz, 0.91H, aromatic H *anti* isomer), 7.16 (m, 2H, aromatic H *anti* isomer), 7.02 (d, *J* = 8.5 Hz, 6H, aromatic H *syn* isomer), 5.06 (s, 2.88H, CH_2_ *syn* isomer), 4.64 (s, 1.07H, CH_2_), 3.81 (s, 6H, °CH_3_) (Figure S12).^13^C NMR (101 MHz, DMSO) δ 168.40 (C=O), 161.02(C=O), 154.32, 144.61, 130.60, 128.95, 127.20, 121.56, 114.84, 108.83, 55.69(CH_3_), 42.65(CH_2_) (Figure S13).

### 2.9. N,N′-2,2′-(2-Oxo-1H-benzo[d]imidazole-1,3(2H)-diyl)bis(4-flourobenzylidene) Acetohydrazide) (***6b***)

Colorless crystals were produced: yield = 402 mg (82%), m.p. >300 °C. ^1^H NMR (400 MHz, DMSO) δ 11.85 (s, 0.52H, NH *anti* isomer), 11.73 (s, 1.48H, NH *syn* isomer), 8.25 (s, 0.47H, CH=N *anti* isomer), 8.05 (s, 1.50H, CH=N *syn* isomer), 7.88–7.80 (m, 3H, aromatic H *syn* isomer), 7.80–7.74 (m, 1H, aromatic H *anti* isomer), 7.29 (t, *J* = 8.4 Hz, 3.61H, aromatic H *syn* isomer),7.11 (m, 4H, aromatic H *syn* isomer), 5.08 (s, 2.98H, CH_2_ *syn* isomer), 4.67 (s, 0.99H, CH_2_ *anti* isomer) (Figure S14).^13^C NMR (101 MHz, DMSO) δ 168.68, 166.59, 163.92, 163.52 (d, *^1^J_CF_* = 247.8 Hz), 143.39, 131.07, 130.22, 129.67 (d, *^3^J_CF_* = 8.5 Hz), 121.41, 116.35 (d, *^3^J_CF_* = 22.6 Hz),108.75 (Figure S15).

### 2.10. N,N′-2,2′-(2-Oxo-1H-benzo[d]imidazole-1,3(2H)-diyl)bis(4-nitrobenzylidene) Acetohydrazide) (***6c***)

Yellow crystals were produced: yield = 505 mg (93%), m.p. > 300 °C.^1^H NMR (400 MHz, DMSO) δ 12.04 (d, *J* = 80.4 Hz, 2H, NH), 8.36 (s, 0.58H, CH=N *anti* isomer), 8.29 (d, *J* = 6.4 Hz, 3.45H, aromatic H), 8.16 (s, 1.57H, CH=N *syn* isomer), 8.04 (d, *J* = 6.3 Hz, 2.66 H, aromatic H *syn* isomer), 7.96 (m, 1.51H, aromatic H *anti* isomer), 7.19 (m, 1.82H, aromatic H), 7.06 (m, 2H, aromatic H), 5.14 (s, 3H, CH_2_, *syn* isomer), 4.72 (s, 1.33H, CH_2_ *anti* isomer) (Figure S16). ^13^C NMR (101 MHz, DMSO) δ 167.53 (C=O), 160.98(C=O), 153.54, 145.43, 133.32, 128.95, 127.20, 120.87, 114.32, 108.55, 42.77(CH2).

### 2.11. Computational Details

The quantum chemical calculations of the studied compounds were carried out using the DFT method (Gaussian 09, Carnegie Mellon University, Gaussian, Inc., Pittsburgh, PA, USA) with the B3LYP functional and 6-31G(d,p) basis set by Gaussian 09 software [44]. The maximum optimization of geometries was done by minimizing the energies corresponding to all the geometrical parameters without changing any molecular symmetry constraints. Gauss View 5.8 [45] was used to draw the frontier molecular orbitals as well as the optimized structure (Figure S2). Calculation of the frequencies indicates the absence of any imaginary frequency modes, which proved the minimum energy of the optimized structures. The gauge including atomic orbital (GIAO) method was done to determine NMR calculations with the same level of theory (Figure S3, Table S3). The 1H isotropic tensors were used as a reference to the TMS calculation at the same level.

### 2.12. In Vitro Cell Culture 

We took two cancer cell lines, human colorectal carcinoma (HCT-116) and human cervical cells (HeLa) supplied by ATCC, Manassas, VA, USA, to study the impact of the compounds on their viability and proliferation. We also included the noncancer cell line, human embryonic kidney cells (HEK-293) supplied by ATCC, Manassas, VA, USA, as a control cell line. The cells were cultured and maintained in DMEM media (Gibco, Waltham, MA, USA), L-glutamine (Gibco, Waltham, MA, USA) (5%), penicillin (Sigma, Burlington, MA, USA) (1%), streptomycin (Gibco, Waltham, MA, USA) (1%), FBS (Invitrogen, Waltham, MA, USA) (10%), and selenium chloride (Gibco, Waltham, MA, USA) (1%) as previously described (1). The cells were grown in 96-well plates in a 5% CO_2_ incubator (Thermo Fisher Scientific, Inc., Waltham, MA, USA) at 37 °C, and 75–80% confluent cells were processed for the MTT assay [46,47].

### 2.13. MTT Assay

The MTT assay was performed as previously described [48,49]. The cells were treated with samples **6a** (3.88 to 97.23 µM), **6b** (4.08 to 102 µM), and **6c** (3.67 to 91.88 µM), respectively. The cells were treated for 48 h and processed to examine cell viability. We included a control group with no added samples. The control cells, embryonic kidney cells (HEK-293), were also treated with samples **6a** (3.88 to 97.23 µM), **6b** (4.08 to 102 µM), and **6c** (3.67 to 91.88 µM). Both the control and the 6a-, 6b-, and 6c-treated samples were treated with 10 µL of MTT (Sigma, Burlington, MA, USA) (5 mg/mL), and further incubated in a CO_2_ incubator for 4 h. After that, the cell culture media was replaced with DMSO (Invitrogen, Waltham, MA, USA) (1%), and the 96-well plates were examined under an ELISA plate reader (Biotek Instruments, Winooski, VT, USA) at a wavelength of 570 nm to calculate the percentage of cell viability.

### 2.14. Apoptotic DAPI Staining

Morphology changes in the nuclei of cancer cells caused by treatment with samples **6a**, **6b**, and **6c** were examined by DAPI staining. Cells were divided into two groups: the control group with no added samples, and the experimental group where samples **6a** (48.61 µM), **6b** (51 µM), and **6c** (45.95 µM) were added. At 48 h after treatment, both groups were exposed to ice-cold (4%) paraformaldehyde and then washed with Triton X-100 in phosphate-buffered saline (PBS). After that, cells were stained with DAPI (Sigma, Burlington, MA, USA) (1.0 μg/mL) for 5 min under a dark environment, washed with PBS, and mounted onto coverslips. DNA staining was examined using Confocal Scanning Microscope (Zeiss, Jena, Germany). The data are presented as the mean (±) standard deviation (SD) obtained from triplicates and were analyzed by one way ANOVA followed by Dennett’s post hoc test with GraphPad Prism Software (GraphPad Software, San Diego, CA, USA).

## 3. Results and Discussion

### 3.1. Ultrasonic Synthesis of 1H-Benzo[d]imidazol-2(3H)-one and Its Derivatives

The 1*H*-Benzo[*d*]imidazol-2(3*H*)-one was effectively synthesized under ultrasonic irradiation in a shorter reaction time of 40 min at 80 °C compared to 6 h under conventional heating [50] or 22 h stirring [51]. 

Many unsuccessful trials for di *N*-alkylation of 1*H*-benzo[*d*]imidazol-2(3*H*)-one with ethyl chloroacetate were carried out using potassium carbonates or sodium carbonates in DMF or acetone under ultrasonic irradiation to promote the reaction. All gave a mixture of mono and dialkylating products even for a longer reaction time of 60 min.

**Scheme 1:**
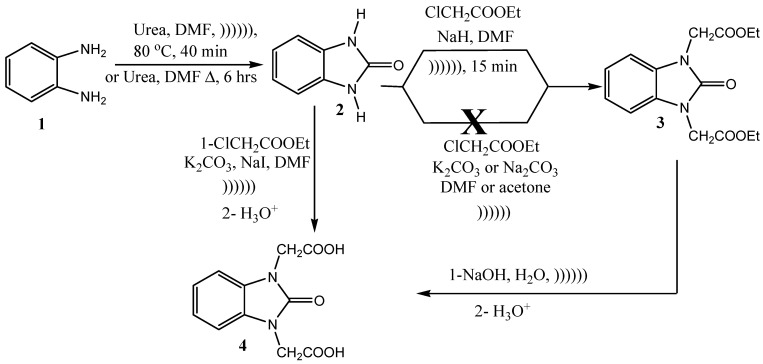
Synthesis of 2,2′-(2-oxo-1*H*-benzo[*d*]imidazole-1,3(2*H*)-diyl)diacetic acid and its ester.

Our attempt to activate the alkyl halide using catalytic amounts of sodium iodide and ultrasonic irradiation led to the reaction proceeding effectively to yield 2,2′-(2-oxo-1*H*-benzo[*d*]imidazole-1,3(2*H*)-diyl)diacetic acid instead of its diethyl ester. This can be explained by the effective ultrasonic dialkylation of the benzimidazolone as well as the basic hydrolysis of the formed ester (Scheme 1).

Diethyl 2,2′-(2-oxo-1*H*-benzo[*d*]imidazole-1,3(2*H*)-diyl)diacetate was effectively synthesized in DMF using sodium hydride as a base under ultrasonic irradiation for only 15 min. The obtained product was characterized using melting points as well as its FTIR (Figures S6 and S7) and UV spectra (Figures S4 and S5) (SHIMADZU, Kyoto, Japan). The dialkylation of the benzimidazolone was confirmed by the integration of the aromatic protons and the methylene of the alkyl chains. The UV spectra of 2,2′-(2-oxo-1*H*-benzo[*d*]imidazole-1,3(2*H*)-diyl)diacetic acid and its diethyl ester were measured in methanol to show λ_max_ at 279 nm, 280 nm for the acid and the ester; respectively.

**Scheme 2:**
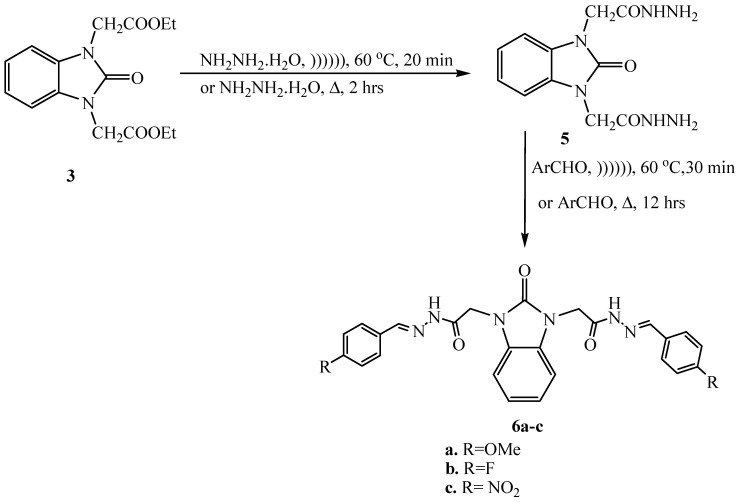
Synthesis of 2,2′-(2-oxo-1H-benzo[d]imidazole-1,3(2H)-diyl)bis(arylidene) acetohydrazide.

The reaction of the diester with hydrazine hydrate effectively produced bis acid hydrazide either under conventional heating or under ultrasonic irradiation, while the reaction needed two hours reflux and only 20 min under ultrasonic irradiation at 60 °C. The condensation reaction of the obtained acid hydrazide with aromatic aldehydes (F, NO_2_ or MeO) produced the corresponding hydrazones with excellent yields (up to 93%). The reaction needed only 30 min irradiation instead of the 12 h for conventional reflux (Scheme 2).

As previously reported, ref. [21] acetylhydrazones can exist in solution as a mixture of (configurational isomers and conformational isomers) *Z-E* and *syn*-*anti*, respectively. The ratio of these isomers is dependent on many factors, including the nature of the solvent and their chelation ability [52]. On the other hand, their X-ray spectra revealed the presence of only the *syn-E* isomer [52,53] where the CO and NH groups permit maximum intermolecular hydrogen bonding. 

The NMR spectra of the prepared acylhydrazones in DMSO confirmed the presence of a mixture of isomers. The signals assigned to the CH_2_CO, CH= and NH groups presented as two signals of each. It has been reported that the signals assigned to the major isomer (*syn*) showed CH_2_CO and CH= groups as downfield and upfield, respectively, with respect to the signals of the minor isomer (*anti*) [54,55]. The ratio of integral intensities of the CH_2_CO and CH= signals was used to calculate the percentage of *anti* and *syn* isomers. The ratio of *syn*:*anti* isomers for CH_3,_ NO_2_, and F derivatives was (0.72:0.28), (0.69:0.31) and (0.75:0.25); respectively.

### 3.2. Optimized Molecular Geometry

**Figure 1:**
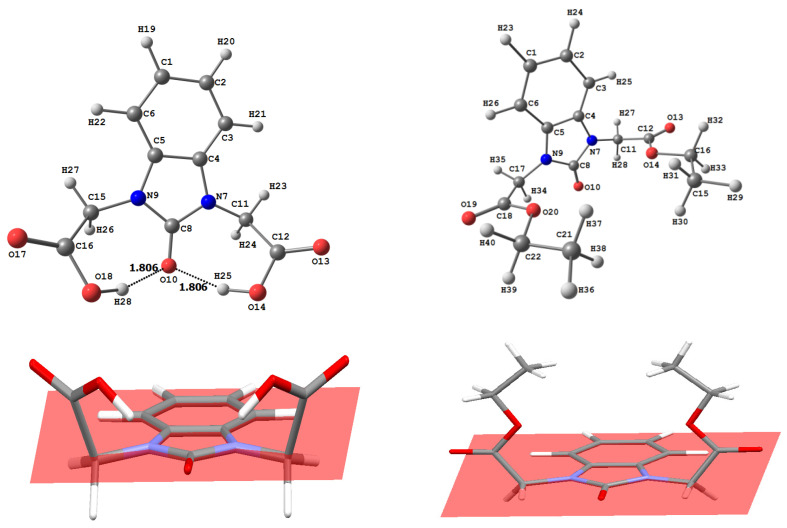
Optimized molecular structure of studied compounds.

The optimized angles and bond lengths obtained for the studied compounds are presented in Table S1. The optimized structures and atom numbering scheme are given in Figure 1. Both molecules have the C1 point group. In the compounds under investigation, the bond distances of C=O of the benzo[*d*]imidazol-2(3*H*)-one moiety were calculated to be 1.252 Å and 1.221 Å for the acid and ester, respectively. These results agree with the X-ray structure of the structurally related ketone (1.213 Å) [56]. It is slightly longer for the acid as the C=O is incorporated in the formation of two intramolecular H-bonding interactions. The acid has two equivalent intramolecular H--O H-bonds with a distance of 1.806 Å. Such interactions do not occur in the ester compound and hence the C=O bond is closer to the experimental value than that for the acid. It seems from Figure 1 that the benzimidazole ring has a planar structure with two carboxylate groups in the *cis*-configuration. The two carboxylate groups are out of the benzo[*d*]imidazol-2(3*H*)-one moiety by about 69° in both molecules.

**Figure 2:**
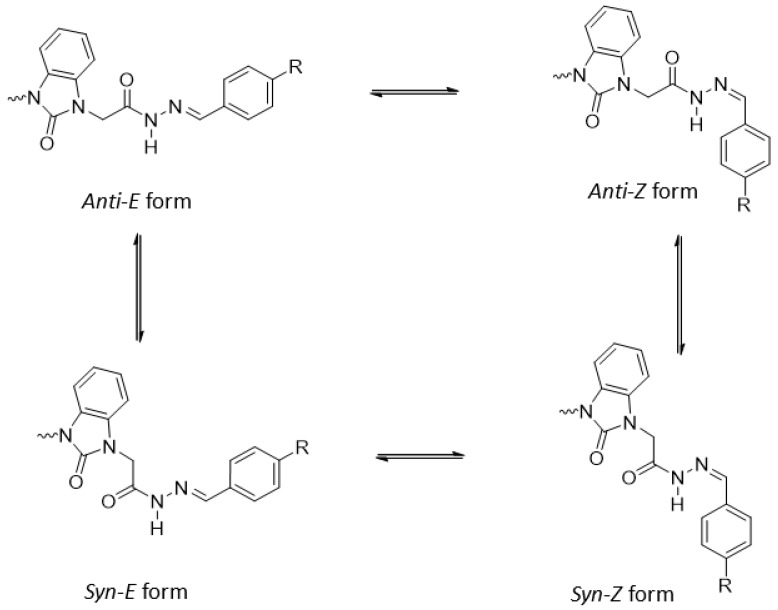
Possible isomers for the prepared acylhydrazones.

Acetohydrazide hydrazones can exist in solution as a mixture of (configurational and conformational isomers) *Z-E* and *syn*-*anti*, respectively. The ratio of these isomers is dependent on many factors, such as the nature of the solvent and their chelation ability [52] (Figure 2 and Figure S1, Table 1 and Table S2).

**Figure 3:**
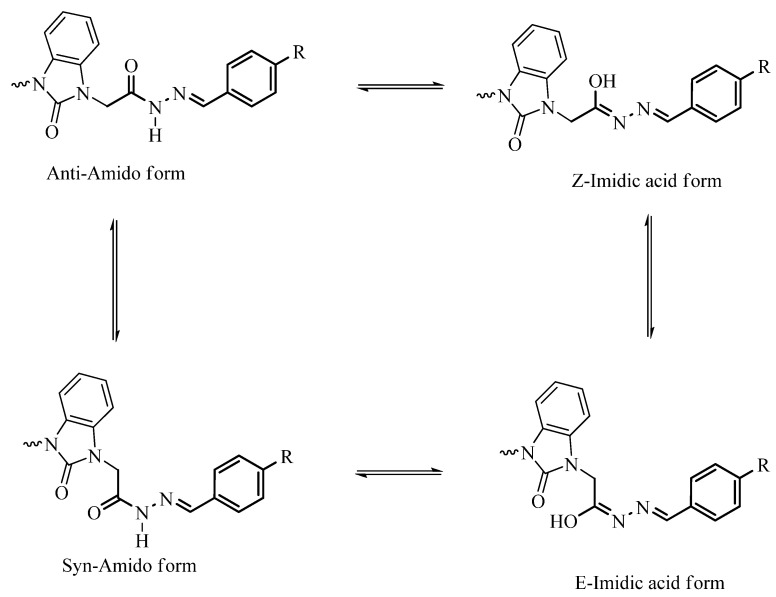
Representation of amido-amidic acid forms of 2,2′-(2-oxo-1*H*-benzo[*d*]imidazole-1,3(2*H*)-diyl)bis(arylideneacetohydrazide).

Based on previous reports that confirm the existence of acyl and aroylhydrazones in polar solvents (such as DMSO solutions) up to 100% as *E*-isomers [56], the *Z*-isomers are excluded in this study. On the other hand, many reports have studied the amide-amidic acid tautomerism of amides and anilides [57,58,59,60,61]. However, to the best of our knowledge, there are no literature reports studying amido-amidic acid tautomerism for acylhydrazones. Considering this kind of tautomerism for the acetohydrazide hydrazones (**6a**–**c**) expands the number of isomers to four for each compound. In addition, amido-amidic acid tautomerism has the additional complexity of also being in *E-Z* for imidic acid forms as well as *syn*-*anti* for amido forms (Figure 3).

To calculate various thermochemical values, geometrical optimization of each tautomer to obtain the minimal energy structure was performed, followed by a frequency calculation at the optimized geometry, Table 1.

The results of the DFT calculations predicted that amido forms are lower energy structures and hence more stable than amidic acid forms by about 28–31 Kcal, and the predominant species is the *syn*-amido tautomer for the methoxy derivative while the anti-amido tautomer is the predominant species for fluoro and nitro derivatives. The energy differences between the two amido forms vary between 0.25 to 1.96 kcal/mol. This small energy difference between the two conformations indicates their coexistence in equilibrium in the gas phase (Table 2).

**Figure 4:**
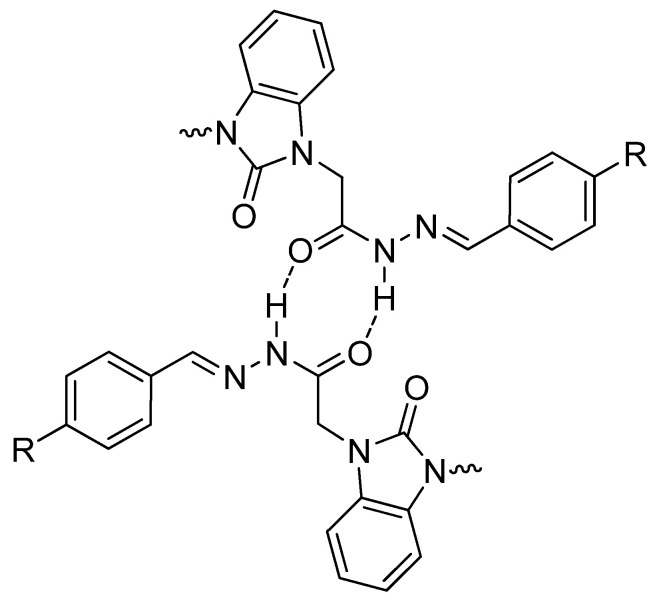
The intermolecular hydrogen bond in the *syn* amido form.

The preference of the *syn*-form could be explained by the *syn* conformation of amide functionality allowing the CO and NH groups to form maximum intermolecular hydrogen bonding (Figure 4) [21,62].

**Figure 5:**
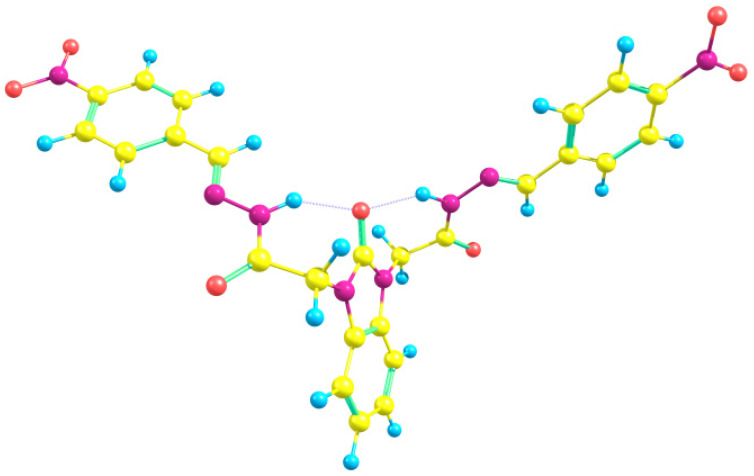
Intramolecular hydrogen bond *anti* amido-tautomer of acylhydrazone **6c**.

On the other hand, the presence of the imidazolone ring carbonyl led to the stabilization of the anti-form due to the creation of an intramolecular hydrogen bond between the NH group and the ring carbonyl oxygen (Figure 5) [21,62].

The arylidene substituent plays an important role in favoring the predominance of either the *syn* or *anti* forms by affecting the strength of the intramolecular hydrogen bond. Where electron attracting substituents such as the fluoro and nitro groups lengthen both the ring carbonyl group, the N-H bond of amide functionality leads to more polarizable donor and acceptor atoms and thus effectively forms intramolecular HB. Hence these derivatives favor the *anti*-form, while electron donating groups such as a methoxy substituent favor the *syn* tautomer.

**Figure 6:**
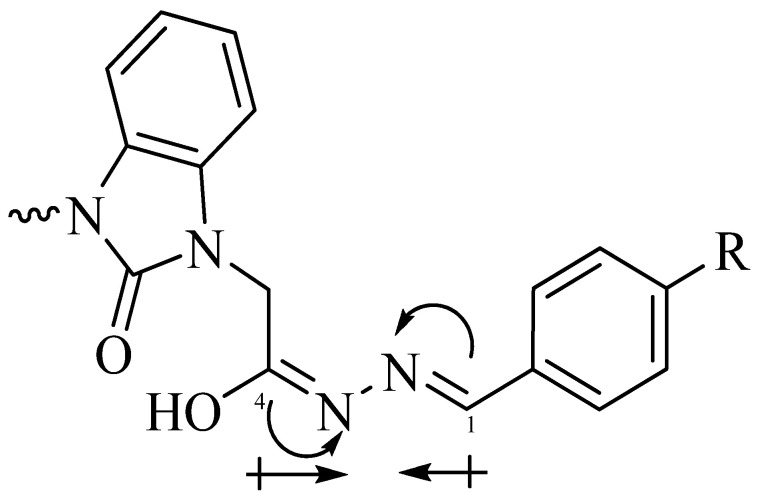
Dipole moment direction of C=N groups in amidic acid form.

Unexpectedly, amidic acid forms are less stable than amido-forms even though they have extra conjugation. This is because of the conjugation extent. When the π electrons in the two imine groups are completely localized, and the orientation of the dipole moments is in opposite directions, these types of resonance do not affect the stability of these compounds [59] (Figure 6).

Moreover, the geometry of some *Z*-amidic isomers enables it to form seven membered intramolecular hydrogen bonds between the OH group and oxygen of the ring carbonyl. However, the geometrical constraints obviously make it less effective and weaker than intermolecular hydrogen bonding with the same donor and acceptor atoms [62,63].

**Figure 7:**
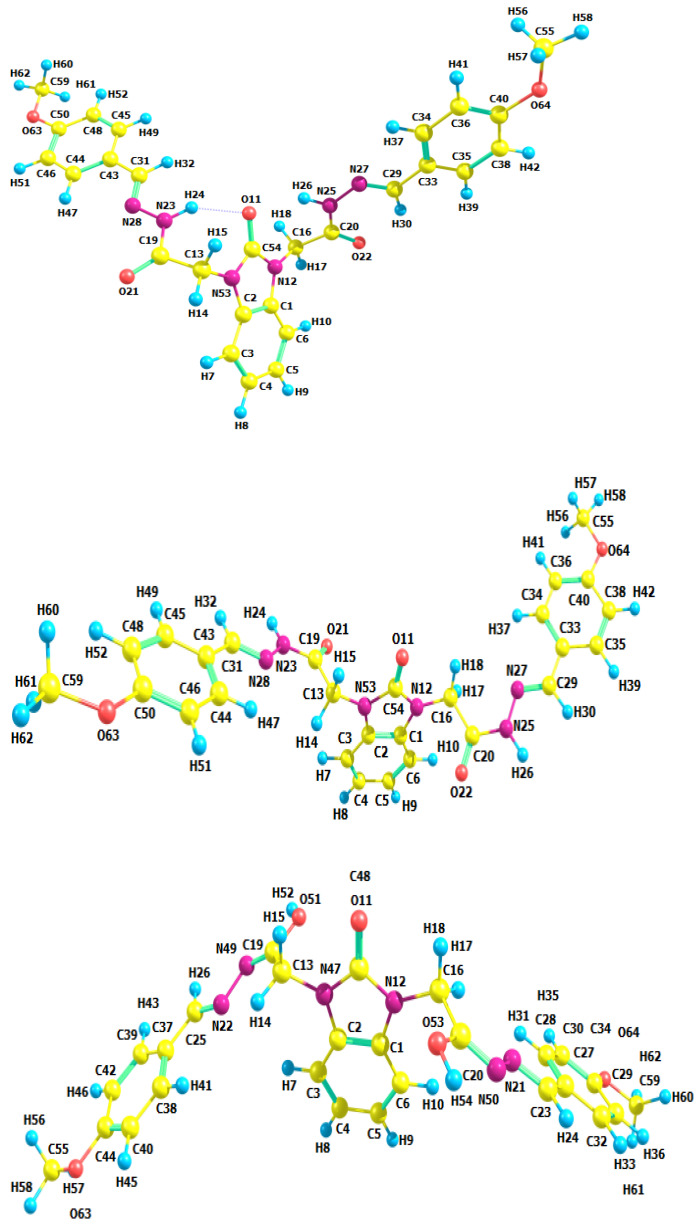

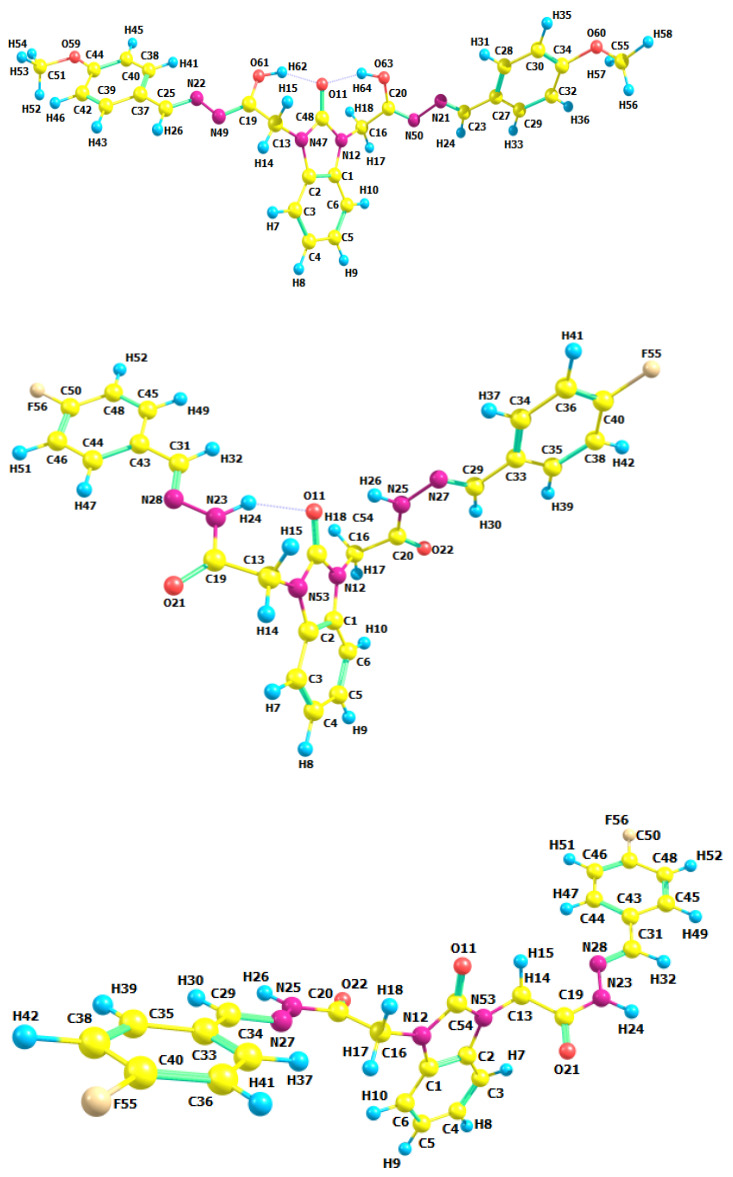

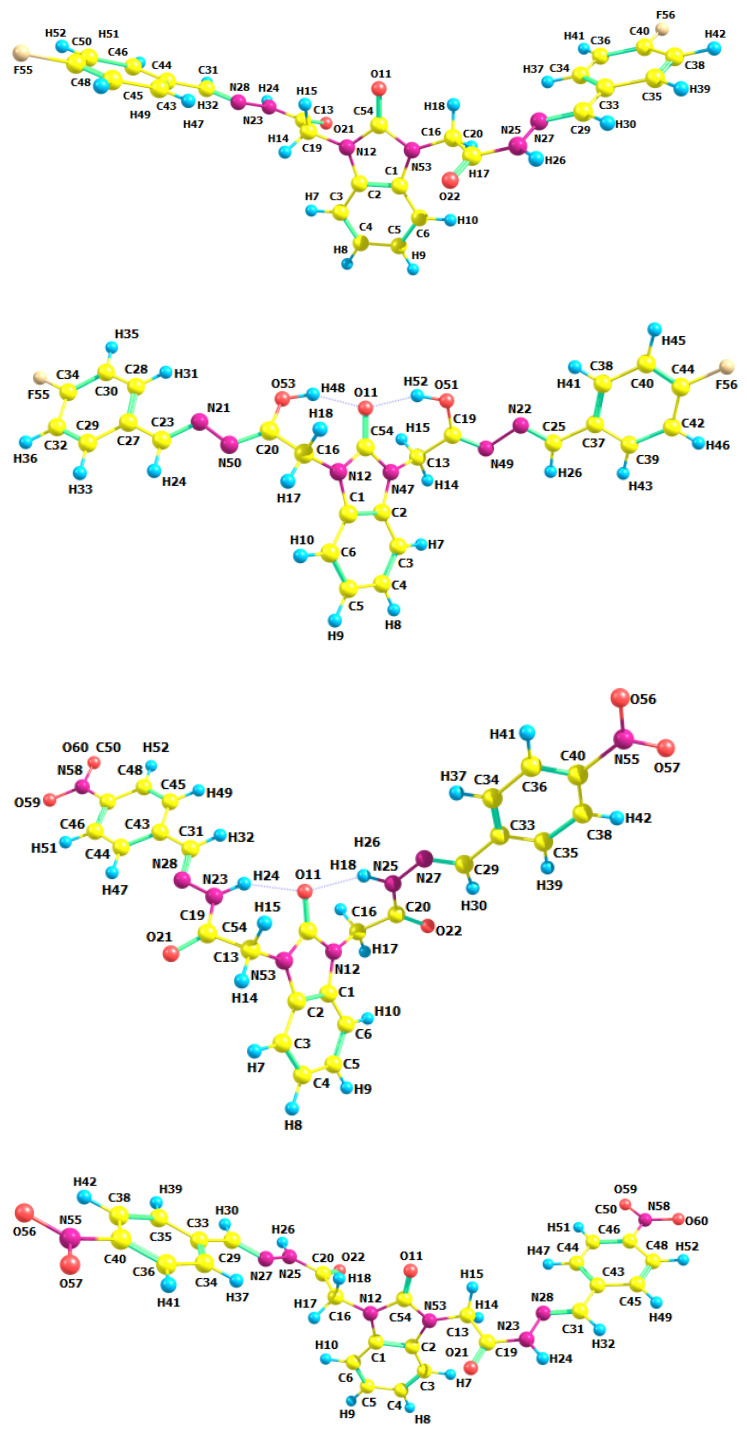

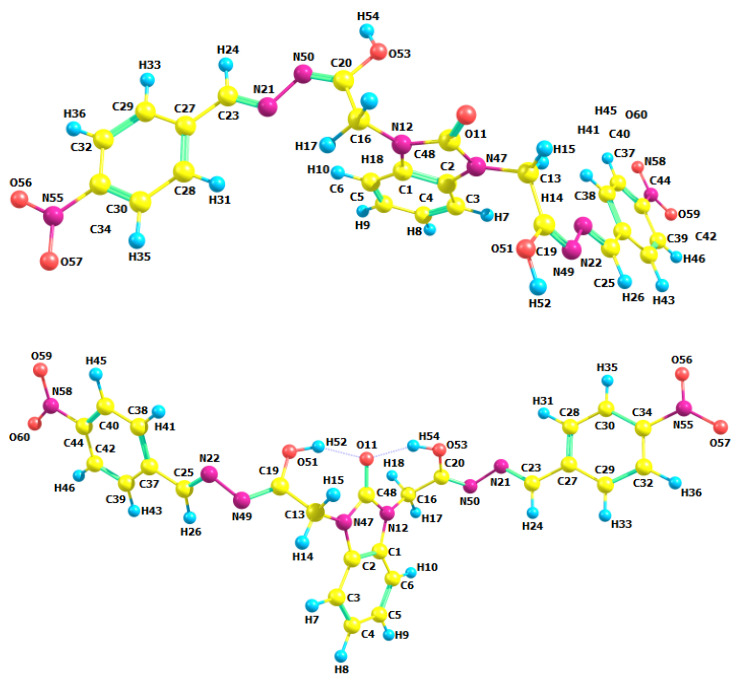
The optimized structures and atom labeling for the tautomeric forms of 2,2′-(2-oxo-1*H*-benzo[*d*]imidazole-1,3(2*H*)-diyl)bis(arylideneacetohydrazide) **6a**–**c**.

The optimized geometrical parameters (bond length, angles and dihedral angles) are listed in Table 3. The numbering of atoms for the optimized geometries of amido-forms of 2,2′-(2-oxo-1*H*-benzo[*d*]imidazole-1,3(2*H*)-diyl)bis(arylideneacetohydrazide) are shown in Figure 7.

The DFT calculations listed in Table 3 show that the ring carbonyl of benzimidazolone in the *syn* tautomer (C54-O11) has a bond length varying between 1.246 to 1.247Å. Moreover, the bond length between the ring carbonyl carbon and nitrogen (N53-C54) varies between 1.399 to 1.400 Å, which indicates that the imidazolone ring is slightly affected by the substituent on the arylidene ring due to its distance. 

In contrast, the bond length of the carbonyl group of the acetohydrazide moiety (C19-O21) is shorter than the ring carbonyl, and varies between 1.240 to 1.245 Å. This could be explained on the basis that the ring carbonyl exists as a carbamide functionality, in which resonance could occur between the lone pair of the adjacent two nitrogen atoms and the carbonyl group, leading to a more single bond character for the carbonyl and hence longer bond length [64].

Obviously, the acetohydrazide carbonyl bond length is affected by the arylidene substituent, as the presence of the strong electron donating methoxy group lengthens it, while the strong electron withdrawing nitro group causes bond shortening. This is consistent with the values of bond length between the carbohydrazide carbonyl carbon and the adjacent nitrogen (C19-N23), where the shortest carbohydrazide carbonyl is accompanied by the longest (C19-N23) and vice versa.

**Figure 8:**
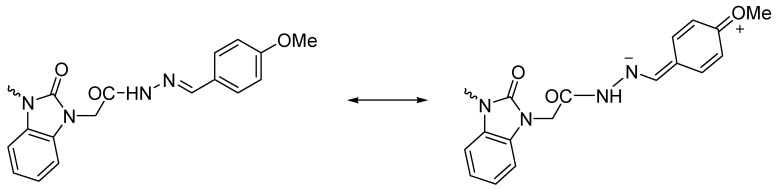
Cross-conjugation between the methoxy group and the imine functionality.

The bond length of the imine functionality (C31-N28) is also affected by the arylidene substituent. In the case of a fluoro or nitro substituent in the *syn* tautomer, the bond length of (C31-N28) is slightly shorter than in the presence of the strong releasing methoxy group. This is in accordance with the cross-conjugation (Figure 8). Cross-conjugation between the methoxy and the imine functionality enhances the polarization of the imine moiety in the right direction towards the more electronegative nitrogen of the imine resulting in lengthening of its bond.

Calculations of bond angles for the studied compounds showed a deviation from the normal trigonal planer for all the carbonyl groups, which confirmed some single bond character for those carbonyls. Moreover, the calculations indicated that the carbonyl carbon of the carbohydrazide moiety is neither in the same plane nor perpendicular to the benzimidazolone ring. Furthermore, the nitrogen of the imine functionality is out of plane of the arylidene ring by 0.59° to 1.03°. These small values are enough to suggest a negligible deviation from coplanarity with the arylidene ring and explain the ability of the imine group to be involved in resonance with the arylidene ring. This is supported by the observed increase in C-N bond length relative to normal Csp^2^-N single bond due to the formation of resonating structures such as those shown in Figure 8.

### 3.3. Antiproliferative Activity

#### 3.3.1. MTT Assay

**Figure 9:**
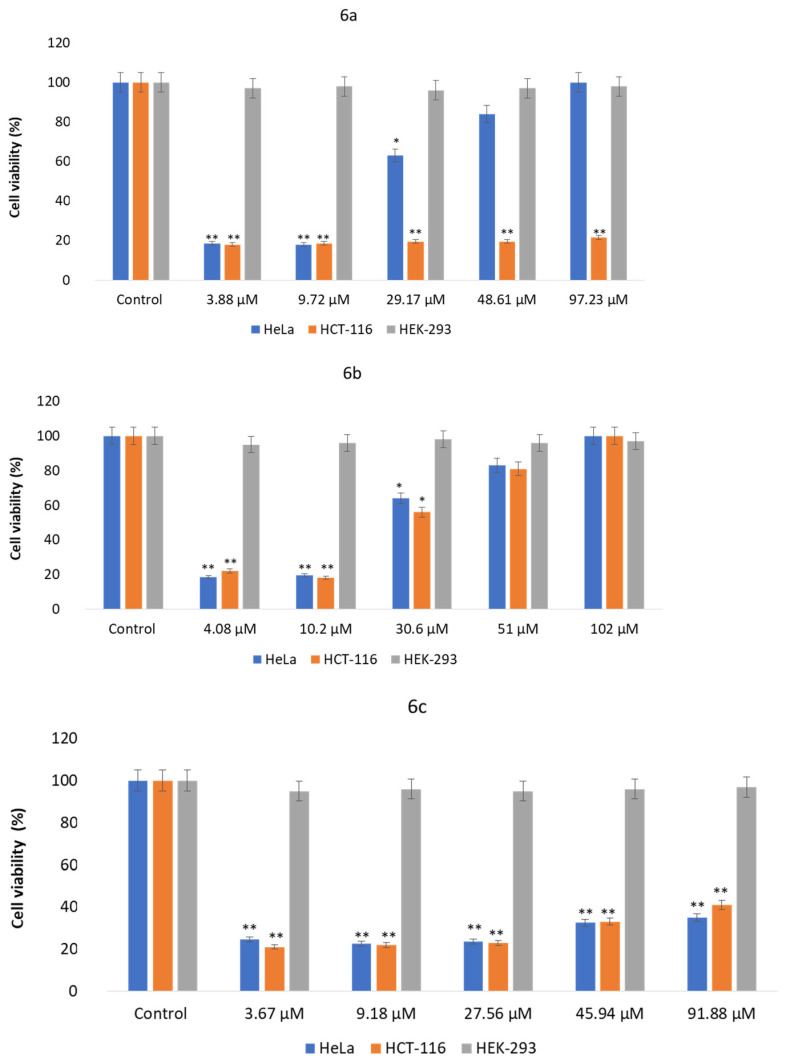
Cell viability with the MTT Assay. Cell viability shows the average impact of samples **6a** (3.88 to 97.23 µM), **6b** (4.08 to 102 µM), and **6c** (3.67 to 91.88 µM) on HeLa cells, HCT-116 cells, and HEK-293 cells 48 h after treatment. * *p* < 0.05; ** *p* < 0.001.

The antiproliferative effect of samples **6a**, **6b**, and **6c** on both colon cancer (HCT-116) and cervical cancer (HeLa) cells was examined in vitro using the MTT assay. The results confirmed a significant decrease in cell viability after the treatments of the cancer cells (HCT-116 and HeLa cells) with samples **6a**, **6b**, and **6c**, especially at low concentrations. Compounds **6a**, **6b**, and **6c** also inhibited human cancer cell growth and proliferation (Figure 9). The IC50 values (Table 4) for HCT-116 were 29.5 + 4.53 µM, 57.9 + 7.01 µM, 40.6 + 5.42 µM for **6a**, **6b** and **6c**, respectively. These results indicate a high capacity of these compounds to inhibit HCT-116 growth when compared, for example, to novel benzimidazole derivatives synthesized by Morcoss et al., which inhibited the proliferation of HCT-116 by approximately 56% at a concentration of 10 mM [1]. Furthermore, some novel pyrazole-benzimidazole derivatives synthesized by Ren et al. have demonstrated significant antiproliferative activity against HCT-116 cells with the IC50 values of 4.33, 5.15 and 4.84 μM [65]. The IC50 values for HeLa cells were 57.1, 65.6 and 33.8 µM for **6a**, **6b** and **6c**, respectively. These results are comparable to the effect of thiourea derivates that inhibit Hela cells with IC50 values in the range of 38–46 µM [66]. Recently, Taherian et al. synthesized a novel quinazolinone derivate that demonstrated a cytotoxic effect against Hela cells with IC50 of 50 µM [67]. The potency of **6a**, **6b** and **6c** to inhibit Hela cells was lower compared to the effect of benzimidazole derivates synthesized recently by Abd El-Meguid et al. with IC50 values ranging from 1.44 to 28.12 µM [68].

Among the synthetized compounds, samples **6a** and **6c** bearing, respectively, MeO and NO_2_ groups presented the highest antiproliferative effect against human colon (HCT-116) and cervical cancer (HeLa) cells, respectively, and thus could be promising anticancer agents. Further study is needed to determine the mechanism of action of these interesting synthetic compounds.

To test the safety of these synthetic compounds, we studied the impact of samples **6a**, **6b** and **6c** on non-cancerous HEK-293 cells, since toxic effects on normal cells is one of the most serious side effects of many anticancer medications. Interestingly, the MTT test confirmed the absence of any significant inhibitory action of the synthetic compounds against HEK-293 cells (Figure 9, Table 4). Thus, the synthesized samples **6a**, **6b** and **6c** possess greater inhibitory effects on HCT-116 and HeLa cells than on HEK-293 cells. This is the first study demonstrating the effect of the synthesized samples **6a**, **6b** and **6c** on the viability of HCT-116 and HeLa cells. We have previously reported the impact of different molecules (nanomaterials and plant extracts) on colon and breast cancer cells [69,70].

#### 3.3.2. Apoptotic DAPI Staining

**Figure 10:**
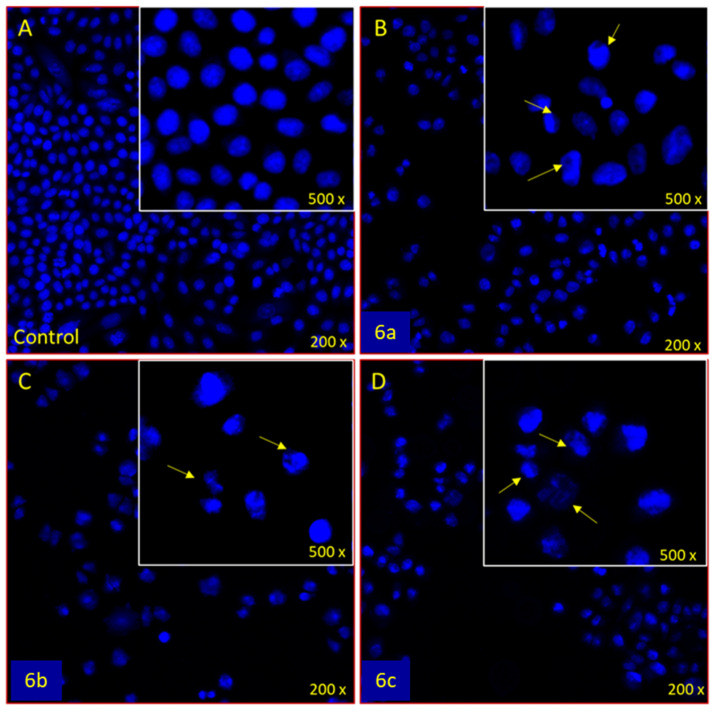
Cancer cell nuclear morphology revealed by DAPI staining; The impact of samples **6a**, **6b**, **6c** on HCT-116 cells stained with DAPI 48 h after treatment. (**A**) Control, (**B**) **6a**, 48.61 µM, (**C**) **6b**, 51 µM, and (**D**) **6c**, 45.95 µM. Arrows show nuclear condensation, shrinkage of nuclei and loss of shape of the nuclei.

**Figure 11:**
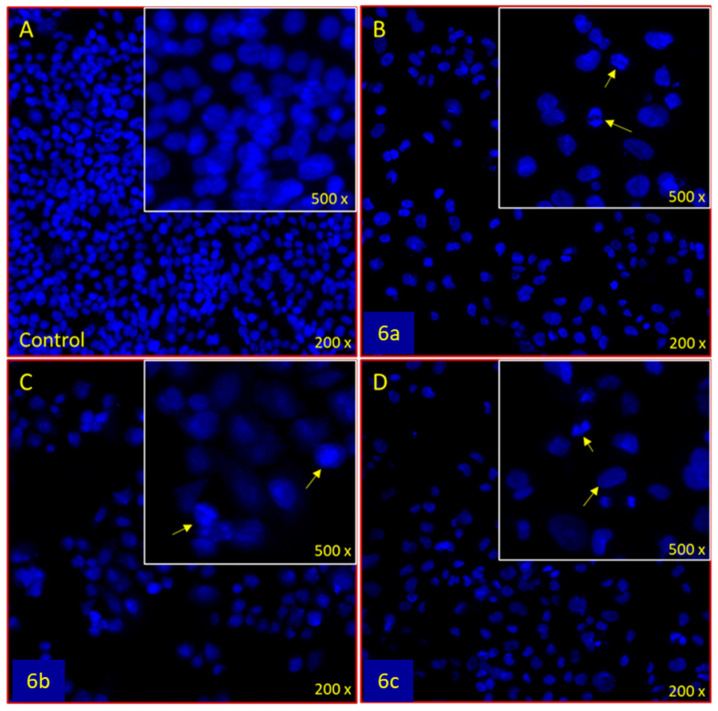
Cancer cell nuclear morphology revealed by DAPI staining; The impact of samples **6a**, **6b**, **6c** on HeLa cells stained with DAPI 48 h after treatment. (**A**) Control, (**B**) **6a**, 48.61 µM, (**C**) **6b**, 51 µM, and (**D**) **6c**, 45.95 µM. Arrows show nuclear condensation, shrinkage of nuclei and loss of shape of the nuclei.

DAPI is a fluorescent dye that stains DNA and can thus be used to detect nuclear damage, which is one of the hallmarks of apoptotic cells [71,72,73]. Treatment with samples **6a**, **6b** and **6c** caused an increase in nuclear condensation, shrinkage of nuclei and loss of shape of the nuclei compared to the control (Figure 10A–D), which suggests an increase in programmed cell death (i.e., apoptosis). We also analyzed the cervical cancer cells (HeLa) by DAPI staining. Treatment with samples **6a**, **6b**, and **6c** caused an increase in nuclear condensation, shrinkage of nuclei and loss of shape of the nuclei compared to the control (Figure 11A–D).

## 4. Conclusions

Benzimidazole has recently achieved widespread attention and become an important ingredient in contemporary medication innovation and medicinal chemistry. Here, ultrasonic irradiation was used as an effective tool for the synthesis of benzimidazole derivatives: diethyl 2,2′-(2-oxo-1*H*-benzo[*d*]imidazole-1,3(2*H*)-diyl)diacetate and its arylideneacetohydrazide derivatives. A complete characterization of the prepared compounds was investigated using NMR, and FTIR spectroscopy. The molecular structure of the studied compounds and their conformational amide isomers and configurational *E-Z* arylidene or amidic acid isomers was investigated using the DFT/B3LYP method and 6-31G(d,p) basis set. The results showed that the energy differences between the two amido forms for all compounds varies between 0.25 to 1.95 kcal/mol, which is evidence of their coexistence. However, the amidic acid forms are excluded because of their high energy difference (29.6 Kcal). The NMR spectra showed that *Syn-E* amido isomers for all compounds had the lowest energy structures among the other isomers. The anticancer activity of the synthetic compounds was studied by MTT assay and DAPI staining. The results revealed that all the synthesized compounds exhibited a strong anticancer activity against both colon cancer (HCT-116) and cervical cancer (HeLa) cells while remaining safe for normal human cells. In the future, we aim to analyze the effects of these interesting benzimidazole derivatives on cell cycle by flow cytometry and investigate their mechanism of action.

## Figures and Tables

**Table 1:** B3LYP calculated thermal-corrected energy, thermodynamic properties, enthalpy (H), free energy (G), ΔE, ΔH, ΔG values for tautomeric forms of compounds **6a**–**c** using the 6-31G basis set.

Parameters		E*_tot_* (hartrees)	ZPVE (hartrees)	E_corr_ (hartrees)	H (hartrees)	G (hartrees)	µ Deby	ΔE (kcal/mol)	ΔH (kcal/mol)	ΔG (kcal/mol)
**6a**	*syn*	−1748.6260	0.507655	−1748.1184	−1748.0833	−1748.1922	3.619	0.50	0.25	1.7
	*anti*	−1748.6271	0.509456	−1748.1176	−1748.0829	−1748.1895	3.031			
	*Z*	−1748.5762	0.506670	−1748.0696	−1748.0348	−1748.1419	6.193	1.20	0.94	1.82
	*E*	−1748.5783	0.506799	−1748.0715	−1748.0363	−1748.1448	5.801			
**6b**	*syn*	−1718.0825	0.425877	−1717.6566	−1717.6251	−1717.7250	0.873	0.56	0.82	0.82
	*anti*	−1718.0852	0.427737	−1717.6575	−1717.6264	−1717.7263	3.721			
	*Z*	−1718.0343	0.424811	−1717.6095	−1717.5782	−1717.6784	7.045	0.25	0.31	1.38
	*E*	−1718.0334	0.424245	−1717.6091	−1717.5777	−1717.6762	2.419			
**6c**	*syn*	−1928.4927	0.446261	−1928.0465	−1928.0116	−1928.1217	4.038	1.69	1.95	0.25
	*anti*	−1928.4972	0.448051	−1928.0492	−1928.0147	−1928.1221	8.282			
	*Z*	−1928.4466	0.445125	−1928.0015	−1927.9669	−1928.0754	11.412	1.44	1.63	0.63
	*E*	−1928.4444	0.445191	−1927.9992	−1927.9643	−1928.0744	0.9046			

**Table 2:** B3LYP calculated ΔE and ΔH values for amido-amidic acid forms of compounds **6a**–**c** using the 6-31G basis set.

	ΔE (kcal/mol)				ΔH (kcal/mol)			
	*Syn-Z*	*Syn-E*	*Anti-Z*	*Anti-E*	*Syn-Z*	*Syn-E*	*Anti-Z*	*Anti-E*
**6a**	30.62	29.93	30.12	28.92	30.43	29.49	30.18	29.24
**6b**	29.56	29.81	30.12	30.37	29.43	29.74	30.25	30.56
**6c**	28.24	29.86	29.93	31.38	28.05	29.68	30.00	31.62

**Table 3:** Selected structure parameters of optimized geometry of amido-forms of compounds **6a**–**c** determined by the B3LYP/6-31G method.

Structural Parameters	6a		6b		6c	
	*Syn*	*Anti*	*Syn*	*Anti*	*Syn*	*Anti*
*R* ^(a)^						
C54-O11	1.246	1.269	1.247	1.270	1.247	1.271
N53-C54	1.400	1.384	1.400	1.385	1.399	1.384
C19-O21	1.243	1.242	1.242	1.241	1.240	1.250
C19-N23	1.381	1.376	1.384	1.378	1.389	1.375
N23-H24	1.019	1.027	1.027	1.027	1.028	1.020
N23-N28	1.378	1.381	1.374	1.378	1.366	1.383
C31-N28	1.297	1.297	1.296	1.296	1.297	1.301
A ^(a)^						
N12-C16-C20	112.14	114.05	112.08	113.92	112.00	113.96
C16-C20-N25	115.41	113.87	115.60	113.92	115.72	112.90
N12-C54-N53	105.68	107.22	105.67	107.26	105.67	107.34
C20-N25-N27	121.82	129.82	121.78	120.54	121.76	129.36
N27-C29-C33	122.78	119.94	122.14	121.291	121.63	119.13
D ^(a)^						
C19-C13-N53-C54	100.18	80.87	99.46	80.09	98.26	79.45
N28-C31-C43-C44	0.75	0.346	1.03	0.22	0.59	0.03
C1-C2-N53-C54	1.30	0.729	1.29	1.12	1.28	1.22

^(a)^*R*, bond length (A^o^); A, bond angle (^o^); D, dihedral angle (^o^).

**Table 4:** IC 50 values of samples (**6a**, **6b**, **6c**) on HCT-116, HeLa, and HEK-293 cells 48 h after treatment.

Compound	HCT-116 (IC50)	HeLa (IC50)	HEK-293 (IC50)
**6a**	29.5 + 4.53 µM	57.1 + 6.7 µM	NI
**6b**	57.9 + 7.01 µM	65.6 + 6.63 µM	NI
**6c**	40.6 + 5.42 µM	33.8 + 3.54 µM	NI

## Data Availability

Not applicable.

## Supplementary Materials

The following are available online at https://www.mdpi.com/article/10.3390/ma15072544/s1, Figure S1: The molecular electrostatic potential of the studied compounds, Figure S2: The ground state isodensity surface plots for the frontier molecular orbitals, Figure S3: The correlation graphs between the calculated and experimental chemical shifts of the studied compounds, Figure S4: UV spectra diethyl N,N-2,2′-(2-oxo-1*H*-benzo[*d*]imidazole-1,3(2*H*)-diyl)diacetate in methanol, Figure S5: UV spectra of N,N-2,2′-(2-oxo-1*H*-benzo[*d*]imidazole-1,3(2*H*)-diyl)diacetic acid in methanol, Figure S6: IR spectra of diethyl N,N-2,2′-(2-oxo-1*H*-benzo[*d*]imidazole-1,3(2*H*)-diyl)diacetate, Figure S7: IR spectra of N,N-2,2′-(2-oxo-1*H*-benzo[*d*]imidazole-1,3(2*H*)-diyl)diacetic acid, Figure S8: ^1^HNMR of diethyl N,N-2,2′-(2-oxo-1*H*-benzo[*d*]imidazole-1,3(2*H*)-diyl)diacetate, Figure S9: ^1^HNMR of N,N-2,2′-(2-oxo-1*H*-benzo[*d*]imidazole-1,3(2*H*)-diyl)diacetic acid, Figure S10: ^1^HNMR of 2,2′-(2-oxo-1*H*-benzo[*d*]imidazole-1,3(2*H*)-diyl)diacetohydrazide, Figure S11: ^13^CNMR of 2,2′-(2-oxo-1*H*-benzo[*d*]imidazole-1,3(2*H*)-diyl)diacetohydrazide, Figure S12: ^1^HNMR of N,N-2,2′-(2-oxo-1*H*-benzo[*d*]imidazole-1,3(2*H*)-diyl)bis(4 methoxybenzylidene)acetohydrazide), Figure S13: ^13^CNMR of N,N-2,2′-(2-oxo-1*H*-benzo[*d*]imidazole-1,3(2*H*)-diyl)bis(4-methoxybenzylidene)acetohydrazide), Figure S14: ^1^HNMR of N,N-2,2′-(2-oxo-1*H*-benzo[*d*]imidazole-1,3(2*H*)-diyl)bis(4 flourobenzylidene)acetohydrazide), Figure S15: ^13^CNMR of N,N-2,2′-(2-oxo-1*H*-benzo[*d*]imidazole-1,3(2*H*)-diyl)bis(4-flourobenzylidene)acetohydrazide), Figure S16: ^1^HNMR of N,N-2,2′-(2-oxo-1*H*-benzo[*d*]imidazole-1,3(2*H*)-diyl)bis(4-nitrobenzylidene)acetohydrazide), Table S1: The calculated bond distance (Å) and angles (°) of the studied compounds using B3LYP/6-31G(d, p) method, Table S2: The natural atomic charges calculated at the B3LYP/6-31G(d,p), Table S3: Calculated and experimental ^1^H-NMR chemical shifts of the studied compounds.
