# Supplementary material for: Synthesis, DFT Molecular Geometry and Anticancer Activity of Symmetrical 2,2′-(2-Oxo-1H-benzo[d]imidazole-1,3(2H)-diyl) Diacetate and Its Arylideneacetohydrazide Derivatives
Source: Materials (Basel). 2022 Mar 30;15(7):2544. doi: 10.3390/ma15072544 (PMC8999490; doi:10.3390/ma15072544)
Supplement: Supplementary file 1 [file materials-15-02544-s001.zip › materials-1578956-supplementary.pdf]

Supplementary Information

Article

Synthesis, DFT molecular geometry and anti-cancer activity of Symmetrical 2,2'-(2-oxo-1*H*-benzo[*d*]imidazole-1,3(2*H*)-diyl) diacetate and its arylideneacetohydrazide derivatives

Manel Dhahri [1], Firdos Alam Khan [2], Abdul-Hamid Emwas [3], Rua B. Alnoman [4], Mariusz Jaremko [5], Nadjet Rezki [6] , Mohamed Reda Aouad [6] and Mohamed Hagar [4, 7,*]

[1] Faculty of Science Yanbu, Biology Department, Taibah University, 46423, Yanbu El-Bahr, Saudi Arabia
[2] Department of Stem Cell Research, Institute for Research and Medical Consultations (IRMC); Imam Abdulrahman Bin Faisal University, P.O. Box 1982, 31441 Dammam-Saudi Arabia
[3] King Abdullah University of Science and Technology. Core Labs. Thuwal 23955-6900, Kingdom of Saudi Arabia
[4] Faculty of Science, Chemistry Department, Taibah University, Yanbu Branch, 46423, Yanbu, Saudi Arabia
[5] King Abdullah University of Science and Technology (KAUST), Biological and Environmental Sciences & Engineering Division (BESE), Thuwal, 23955-6900, Saudi Arabia
[6] Department of Chemistry, College of Science, Taibah University, Al-Madinah Al-Munawarah 30002, Saudi Arabia,
[7] Faculty of Science, Chemistry Department, Alexandria University, Alexandria, Egypt.
* Correspondence: Mohamedhaggar@gmail.com (Mohamed Hagar)

Natural charge

The important properties of molecular structures such as molecular polarizability, dipole moment and electronic structure of a compound depend on the distribution of atomic charge. Table S2. shows the natural atomic charges calculated at different atomic sites. The O- and N-atoms of the compounds under study have electronegative nature where the O-atoms are more electronegative than N-atoms. In both compounds, the two N-atoms of the benzo[*d*]imidazole moiety are equivalent. Both compounds are polar where the calculated dipole moments of the acid and ester are 7.2003 and 3.3865 Debye, respectively; hence the acid is more polar than ester. For the acid, the most electropositive H-sites are those of the COOH groups which agree with the high acidic character of these sites.

Molecular electrostatic potential (MEP) map is an evidence to predict the electrophilic and nucleophilic reactive sites, and to study biological recognition and hydrogen bonding interactions [25,26]. Moreover, it gives an idea about the charge distribution and charge related properties of molecules. Figure 2 shows the MEP figures of both compounds that calculated by B3LYP method with 6–31G(d,p) basis set. Figure S1 indicates that the negative regions (red) are mainly localized over the O-atoms of the carbonyl groups. Hence, these atoms of the studied compounds are the most proper sites to attack the positive regions and the most reactive sites for electrophilic attack. On the other hand, the maximum positive regions (blue) are localized over the C-atom of carbonyl groups of the acid and H-atoms of the ester. these two sites are the most reactive sites for nucleophilic attack.

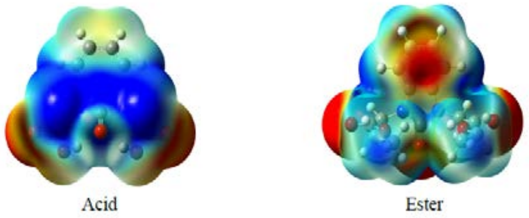

Figure S1. The molecular electrostatic potential of the studied compounds.

Frontier molecular orbitals

The energy and electron densities of the frontier molecular orbitals (FMOs) are very important features for physicists and chemists [30]. Where, the level of energy of the highest occupied molecular orbital (HOMO) and the lowest unoccupied molecular orbital (LUMO) as well as the amount of the energy gap reflect the reactivity of molecule in chemical reactions. Nevertheless, the HOMO-LUMO energy gap is an indicator for the bioactivity from intramolecular charge transfer (ICT) [27,28]. The energy gap (ΔE) between HOMO–LUMO is the lowest energy ICT. The E_{HOMO} and E_{LUMO} of the acid and its ester are calculated using the B3LYP/6–31G(d,p) method. Figure S2. shows the HOMO and LUMO views of the studied compounds. The HOMO–LUMO energy gaps (ΔE) are 5.6124 (221 nm) and 5.4393 eV (228 nm) for the acid and ester, respectively. This intramolecular charge transfer belongs mainly to π–π* excitations as the HOMO and LUMO of both compounds are delocalized over the π-system of benzo[*d*]imidazole moiety. Experimentally, λ_{max} in methanol as solvent at longer wave length of 279 and 280 nm for the acid and ester, respectively. This bathochromic shift in observed λ_{max} experimentally compared to the gas phase calculations could be attributed to solvent-solute interactions. Moreover, the molar absorpitivity (ε) were 4.11 * 10^3 $M^{-1}cm^{-1}$ for the ester and 6.79 * 10^3 $M^{-1}cm^{-1}$ for the acid. The increase in ε of the acid is most probably due to the ability of the acid to form hydrogen bonding**.**

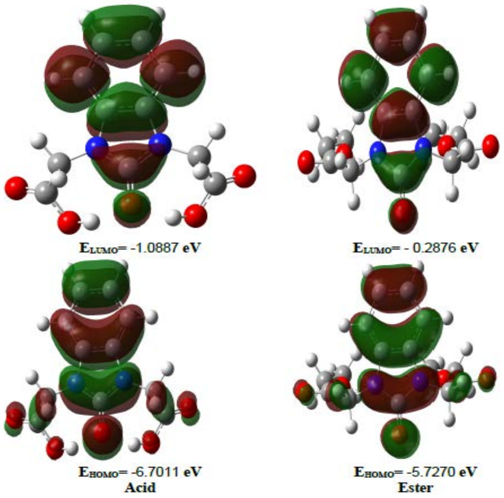

Figure S2. The ground state isodensity surface plots for the frontier molecular orbitals.

Table S1. The calculated bond distance (Å) and angles (˚) of the studied compounds using B3LYP/6-31G(d, p) method.

**Acid**				**Ester**			
R(1-2)	1.400	A(4-3-21)	121.7	R(1-2)	1.397	A(4-5-9)	107.0
R(1-6)	1.398	A(3-4-5)	121.4	R(1-6)	1.402	A(4-7-8)	110.2
R(1-19)	1.085	A(3-4-7)	131.8	R(1-23)	1.085	A(4-7-11)	127.5
R(2-3)	1.398	A(5-4-7)	106.8	R(2-3)	1.402	A(6-5-9)	131.8
R(2-20)	1.085	A(4-5-6)	121.4	R(2-24)	1.085	A(5-6-26)	121.4
R(3-4)	1.390	A(4-5-9)	106.8	R(3-4)	1.388	A(5-9-8)	110.2
R(3-21)	1.084	A(4-7-8)	109.2	R(3-25)	1.085	A(5-9-17)	127.5
R(4-5)	1.408	A(4-7-11)	127.9	R(4-5)	1.410	A(8-7-11)	122.3
R(4-7)	1.402	A(6-5-9)	131.8	R(4-7)	1.393	A(7-8-9)	105.6
R(5-6)	1.390	A(5-6-22)	121.7	R(5-6)	1.388	A(7-8-10)	127.2
R(5-9)	1.402	A(5-9-8)	109.2	R(5-9)	1.393	A(7-11-12)	115.8
R(6-22)	1.084	A(5-9-15)	127.9	R(6-26)	1.085	A(7-11-27)	111.3
R(7-8)	1.372	A(8-7-11)	122.8	R(7-8)	1.395	A(7-11-28)	106.4
R(7-11)	1.460	A(7-8-9)	107.9	R(7-11)	1.440	A(9-8-10)	127.2
R(8-9)	1.372	A(7-8-10)	126.0	R(8-9)	1.395	A(8-9-17)	122.3

R(8-10)	1.252	A(7-11-12)	114.4	R(8-10)	1.221	A(9-17-18)	115.7
R(9-15)	1.460	A(7-11-23)	108.6	R(9-17)	1.440	A(9-17-34)	106.4
R(11-12)	1.542	A(7-11-24)	108.5	R(11-12)	1.528	A(9-17-35)	111.3
R(11-23)	1.088	A(9-8-10)	126.0	R(11-27)	1.094	A(12-11-27)	107.1
R(11-24)	1.096	A(8-9-15)	122.8	R(11-28)	1.095	A(12-11-28)	107.8
R(12-13)	1.206	A(8-10-25)	100.8	R(12-13)	1.212	A(11-12-13)	122.5
R(12-14)	1.339	A(8-10-28)	100.8	R(12-14)	1.341	A(11-12-14)	112.5
R(14-25)	0.985	A(9-15-16)	114.5	R(14-16)	1.450	A(27-11-28)	108.1
R(15-16)	1.542	A(9-15-26)	108.5	R(15-16)	1.515	A(13-12-14)	124.9
R(15-26)	1.096	A(9-15-27)	108.6	R(15-29)	1.094	A(12-14-16)	115.7
R(15-27)	1.088	A(12-11-23)	107.7	R(15-30)	1.093	A(14-16-15)	107.5
R(16-17)	1.206	A(12-11-24)	108.9	R(15-31)	1.094	A(14-16-32)	108.6
R(16-18)	1.339	A(11-12-13)	121.4	R(16-32)	1.094	A(14-16-33)	108.6
R(18-28)	0.985	A(11-12-14)	115.8	R(16-33)	1.094	A(16-15-29)	109.7
R(10-25)	1.806	A(23-11-24)	108.6	R(17-18)	1.528	A(16-15-30)	110.9
R(10-28)	1.806	A(13-12-14)	122.7	R(17-34)	1.095	A(16-15-31)	110.9
A(2-1-6)	121.4	A(12-14-25)	111.4	R(17-35)	1.094	A(15-16-32)	112.3
A(2-1-19)	119.5	A(14-25-10)	161.0	R(18-19)	1.212	A(15-16-33)	112.3
A(1-2-3)	121.4	A(16-15-26)	108.9	R(18-20)	1.341	A(29-15-30)	108.4
A(1-2-20)	119.5	A(16-15-27)	107.7	R(20-22)	1.450	A(29-15-31)	108.4
A(6-1-19)	119.1	A(15-16-17)	121.4	R(21-22)	1.515	A(30-15-31)	108.4
A(1-6-5)	117.3	A(15-16-18)	115.8	R(21-36)	1.094	A(32-16-33)	107.6
A(1-6-22)	121.1	A(26-15-27)	108.6	R(21-37)	1.094	A(18-17-34)	107.8
A(3-2-20)	119.1	A(17-16-18)	122.7	R(21-38)	1.093	A(18-17-35)	107.1
A(2-3-4)	117.3	A(16-18-28)	111.4	R(22-39)	1.094	A(17-18-19)	122.5
A(2-3-21)	121.1	A(18-28-10)	161.0	R(22-40)	1.094	A(17-18-20)	112.5
		A(25-10-28)	106.2	A(2-1-6)	121.2	A(34-17-35)	108.1
				A(2-1-23)	119.6	A(19-18-20)	124.9
				A(1-2-3)	121.2	A(18-20-22)	115.7
				A(1-2-24)	119.6	A(20-22-21)	107.5
				A(6-1-23)	119.1	A(20-22-39)	108.6
				A(1-6-5)	117.5	A(20-22-40)	108.6
				A(1-6-26)	121.1	A(22-21-36)	109.7
				A(3-2-24)	119.1	A(22-21-37)	110.9
				A(2-3-4)	117.5	A(22-21-38)	110.9
				A(2-3-25)	121.1	A(21-22-39)	112.3
				A(4-3-25)	121.4	A(21-22-40)	112.3
				A(3-4-5)	121.3	A(36-21-37)	108.4
				A(3-4-7)	131.8	A(36-21-38)	108.4
				A(5-4-7)	107.0	A(37-21-38)	108.4
				A(4-5-6)	121.3	A(39-22-40)	107.6

Table S2. The natural atomic charges calculated at the B3LYP/6-31G(d,p).

**Acid**				**Ester**			
C1	-0.2345	C15	-0.3757	C1	-0.2457	C21	-0.7121
C2	-0.2345	C16	0.8124	C2	-0.2457	C22	-0.1209
C3	-0.2577	O17	-0.5683	C3	-0.2716	H23	0.2437
C4	0.1466	O18	-0.7109	C4	0.1518	H24	0.2437
C5	0.1466	H19	0.2513	C5	0.1518	H25	0.2483
C6	-0.2577	H20	0.2513	C6	-0.2716	H26	0.2483

N7	-0.4293	H21	0.2545	N7	-0.4487	H27	0.2642
C8	0.8297	H22	0.2545	C8	0.8362	H28	0.2960
N9	-0.4293	H23	0.2743	N9	-0.4487	H29	0.2440
O10	-0.7252	H24	0.2692	O10	-0.6444	H30	0.2430
C11	-0.3757	H25	0.5158	C11	-0.3616	H31	0.2417
C12	0.8124	H26	0.2692	C12	0.8192	H32	0.2296
O13	-0.5683	H27	0.2743	O13	-0.5973	H33	0.2305
O14	-0.7109	H28	0.5158	O14	-0.5500	H34	0.2959
				C15	-0.7121	H35	0.2642
				C16	-0.1210	H36	0.2440
				C17	-0.3615	H37	0.2418
				C18	0.8192	H38	0.2430
				O19	-0.5973	H39	0.2306
				O20	-0.5500	H40	0.2296

NMR Spectra

The values of isotropic magnetic shielding (IMS) are calculated by the GIAO approach at the 6-31G(d,p) level that were used to predict the ^{13}C- and ^{1}H-NMR chemical shifts (δ_{calc}) for the studied compound, and the results were correlated with the experimental NMR data (δ_{exp}) in DMSO solvent. Table S3. shows the experimental and theoretical ^{1}H-NMR chemical shifts of the acid and ester. According to the results, the calculated chemical shifts were in agreement with the experimental findings. As shown in Figure 4, there is compliance between the experimental and calculated chemical shifts (R^2 =0.977-0.996) (Figure S3).

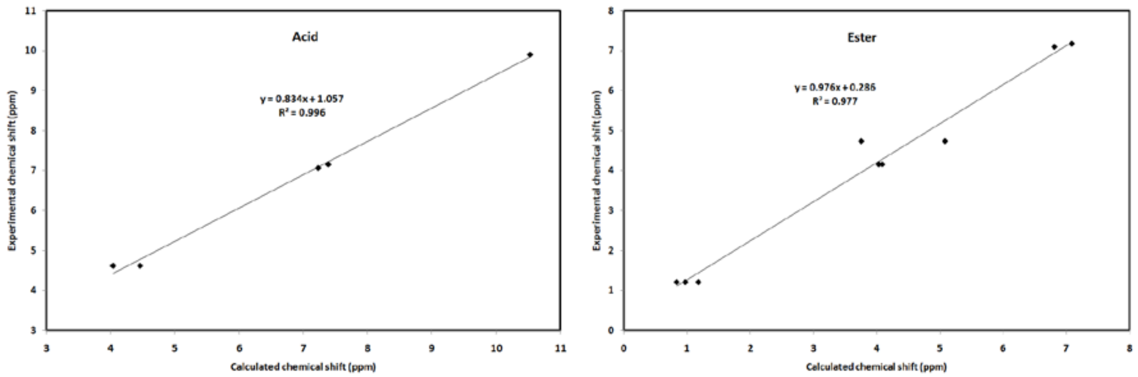

Figure S3. The correlation graphs between the calculated and experimental chemical shifts of the studied compounds.

Table S3. Calculated and experimental ^{1}H-NMR chemical shifts of the studied compounds

**Acid**			**Ester**		
Atom	Calc	Exp	Atom	Calc	Exp
H 19	7.38911	7.16	H 23	7.08991	7.17
H 20	7.38921	7.16	H 24	7.08951	7.17
H 21	7.23021	7.07	H 25	6.81231	7.09
H 22	7.23011	7.07	H 26	6.81451	7.09
H 23	4.03701	4.62	H 27	3.75651	4.73
H 24	4.45621	4.62	H 28	5.08221	4.73
H 25	10.52241	9.90	H 29	0.83381	1.20
H 26	4.45581	4.62	H 30	1.17611	1.20
H 27	4.03741	4.62	H 31	0.96831	1.20
H 28	10.52441	9.90	H 32	4.03711	4.15
			H 33	4.08691	4.15
			H 34	5.08051	4.73

			H 35	3.75541	4.73
			H 36	0.83421	1.20
			H 37	0.97131	1.20
			H 38	1.17911	1.20
			H 39	4.09171	4.15
			H 40	4.03081	4.15

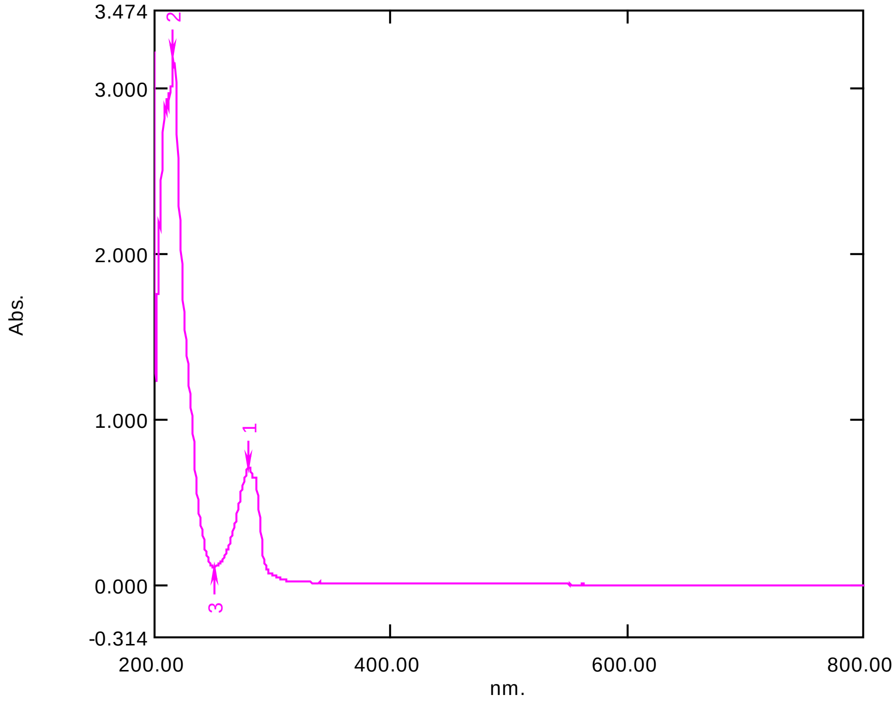

Figure S4. UV spectra diethyl N,N-2,2'-(2-oxo-1*H*-benzo[*d*]imidazole-1,3(2*H*)-diyl)diacetate in methanol.

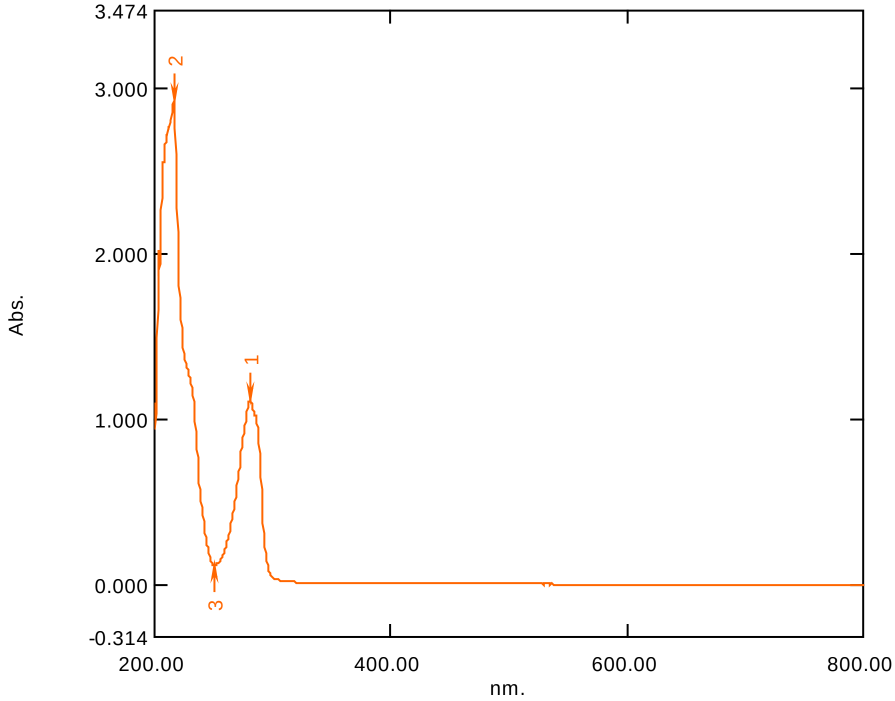

Figure S5. UV spectra of N,N-2,2'-(2-oxo-1*H*-benzo[*d*]imidazole-1,3(2*H*)-diyl)diacetic acid in methanol.

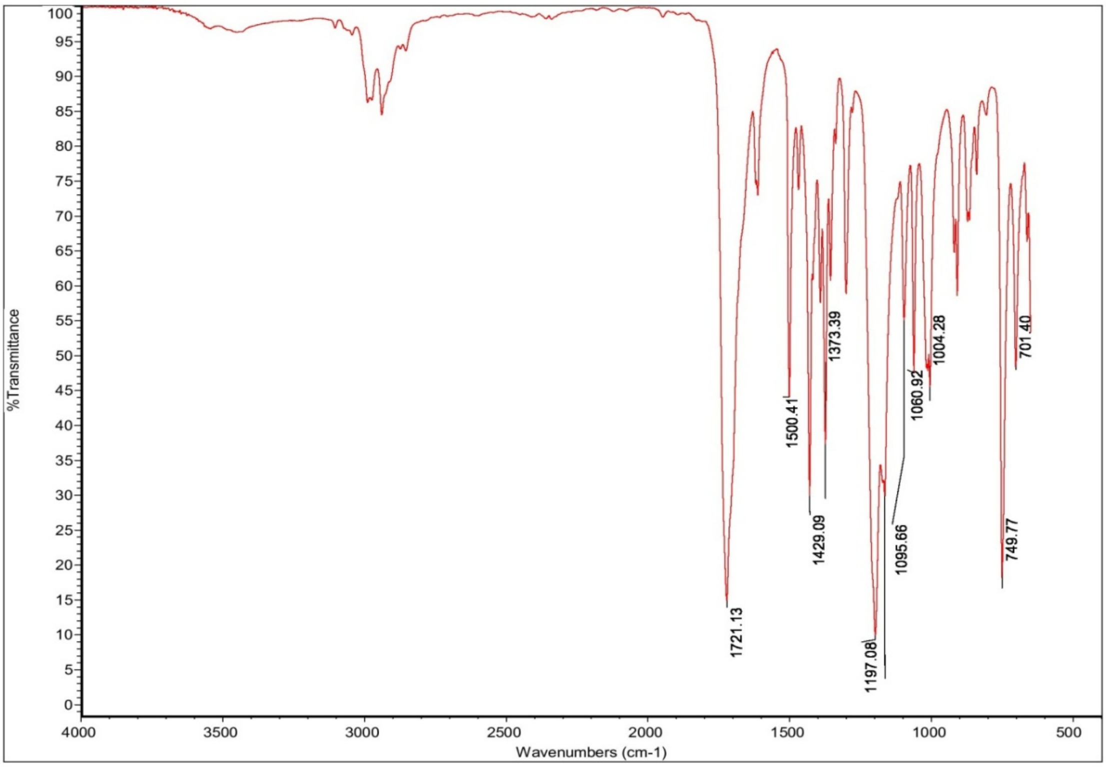

Figure S6. IR spectra of diethyl N,N-2,2'-(2-oxo-1*H*-benzo[*d*]imidazole-1,3(2*H*)-diyl)diacetate.

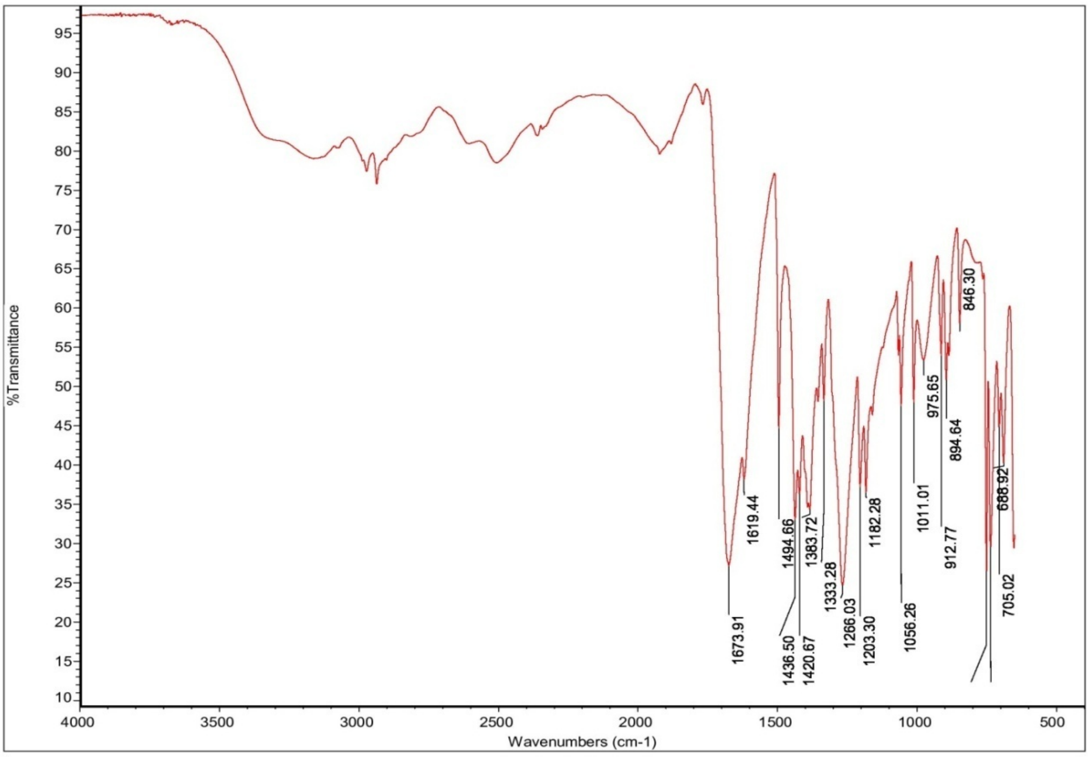

Figure S7. IR spectra of N,N-2,2'-(2-oxo-1*H*-benzo[*d*]imidazole-1,3(2*H*)-diyl)diacetic acid.

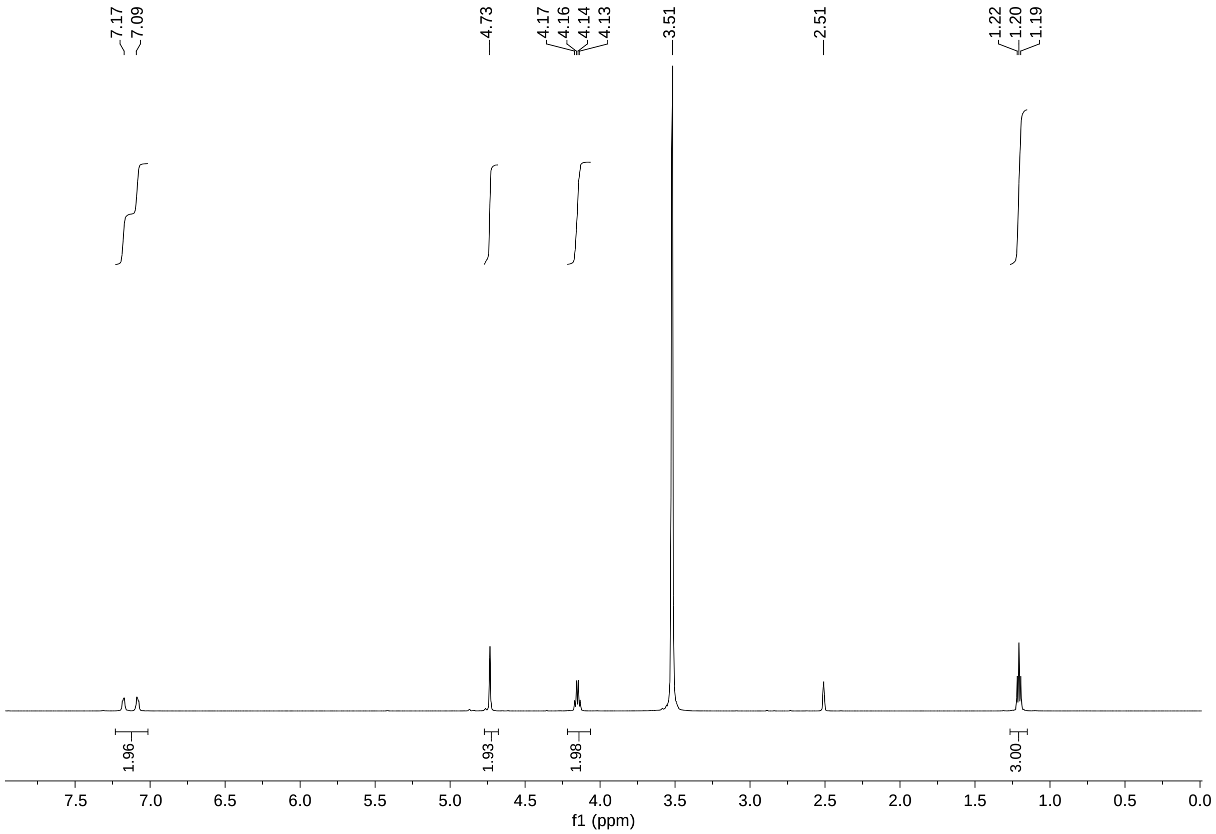

Figure S8. ^{1}HNMR of diethyl N,N-2,2'-(2-oxo-1*H*-benzo[*d*]imidazole-1,3(2*H*)-diyl)diacetate.

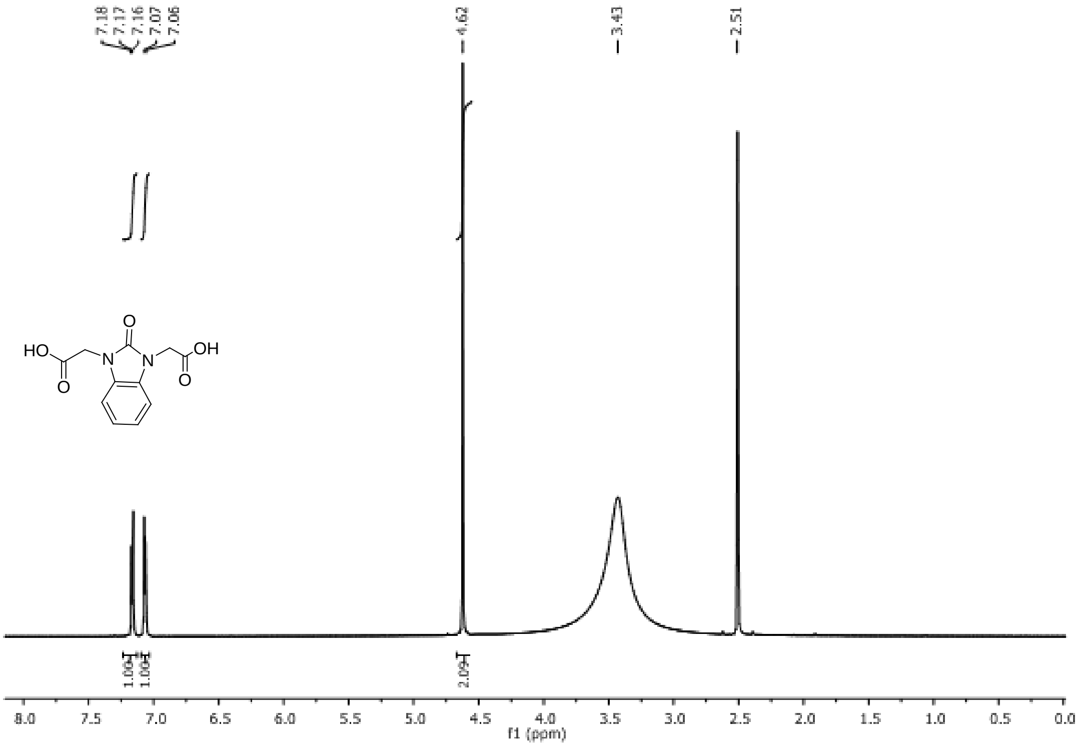

Figure S9. ^{1}HNMR of N,N-2,2'-(2-oxo-1*H*-benzo[*d*]imidazole-1,3(2*H*)-diyl)diacetic acid.

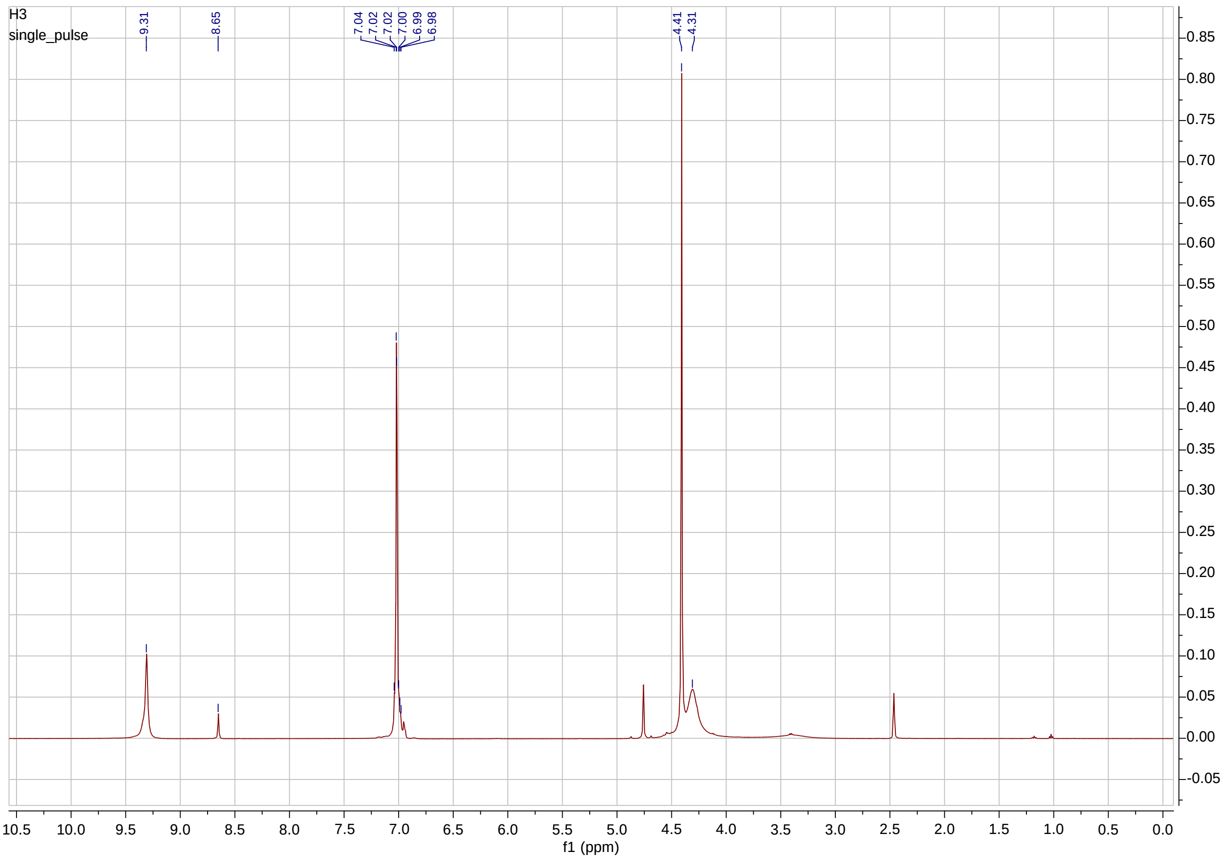

Figure S10. ^{1}HNMR of 2,2'-(2-oxo-1*H*-benzo[*d*]imidazole-1,3(2*H*)-diyl)diacetohydrazide.

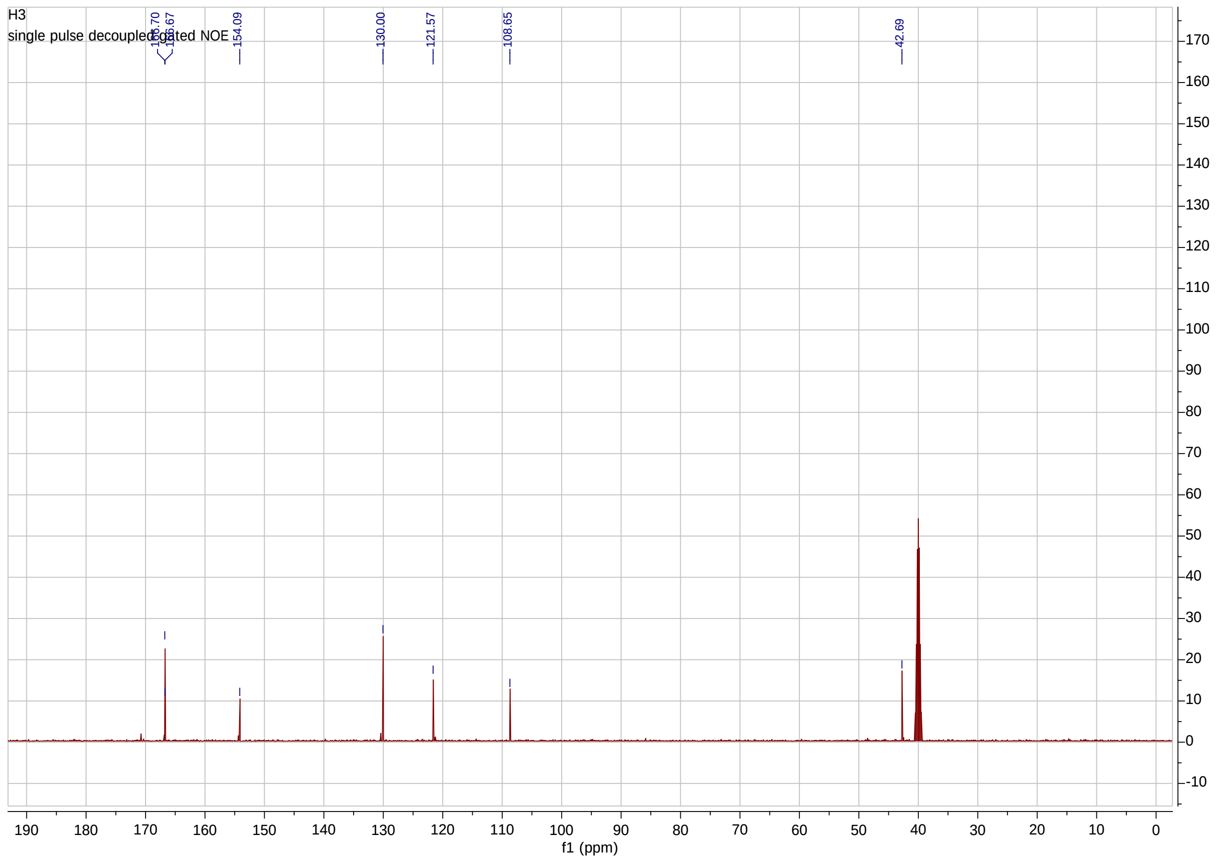

Figure S11. ^{13}CNMR of 2,2'-(2-oxo-1*H*-benzo[*d*]imidazole-1,3(2*H*)-diyl)diacetohydrazide.

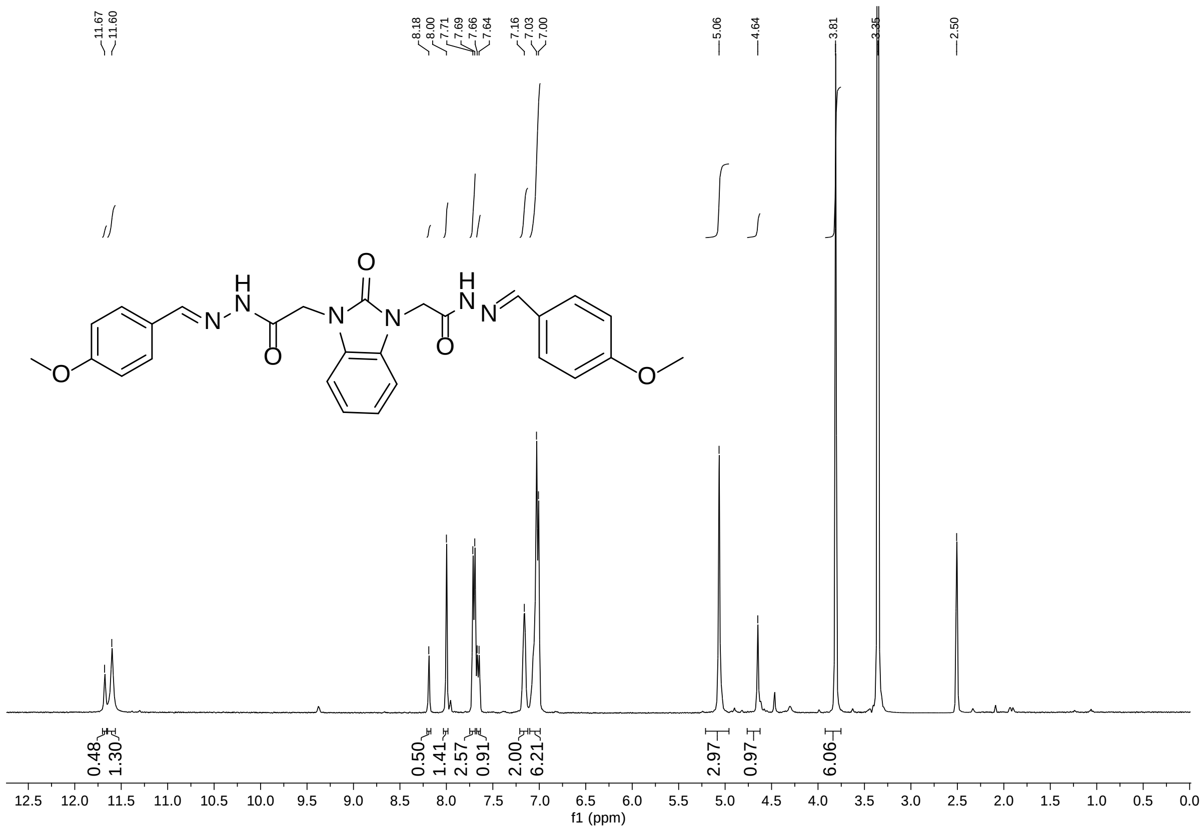

Figure S12. ^{1}HNMR of N,N-2,2'-(2-oxo-1*H*-benzo[*d*]imidazole-1,3(2*H*)-diyl)bis(4 methoxybenzylidene)acetohydrazide).

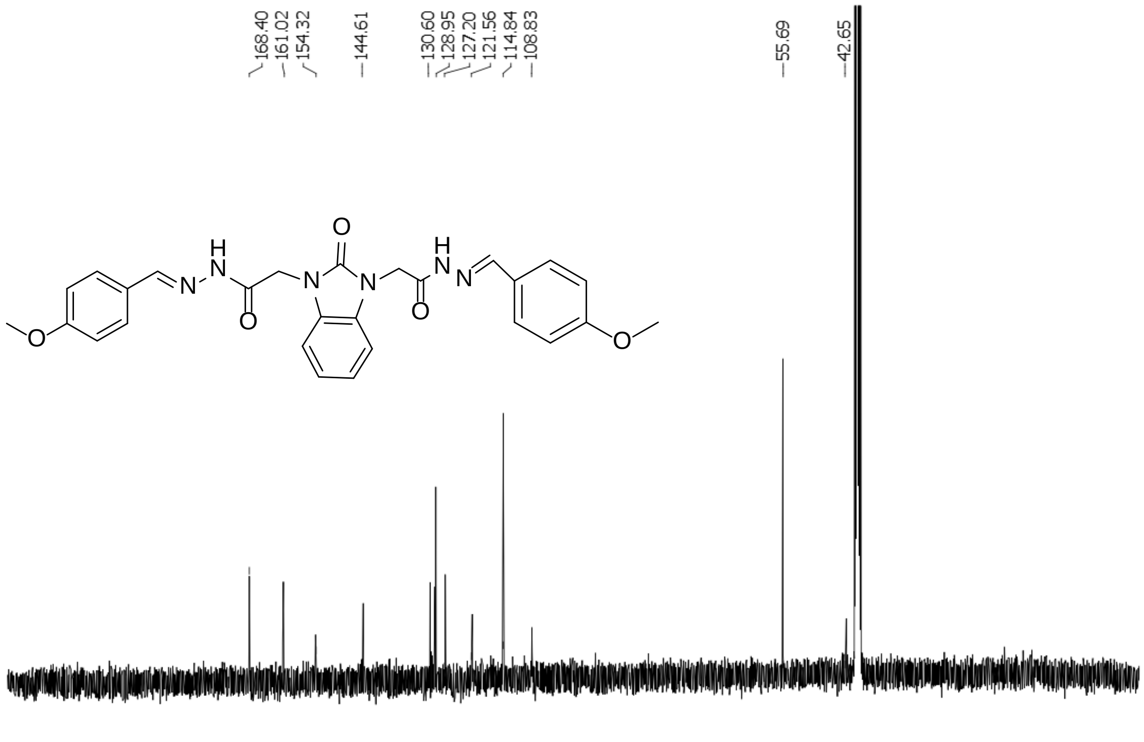

Figure S13. ^{13}CNMR of N,N-2,2'-(2-oxo-1*H*-benzo[*d*]imidazole-1,3(2*H*)-diyl)bis(4-methoxybenzylidene)acetohydrazide).

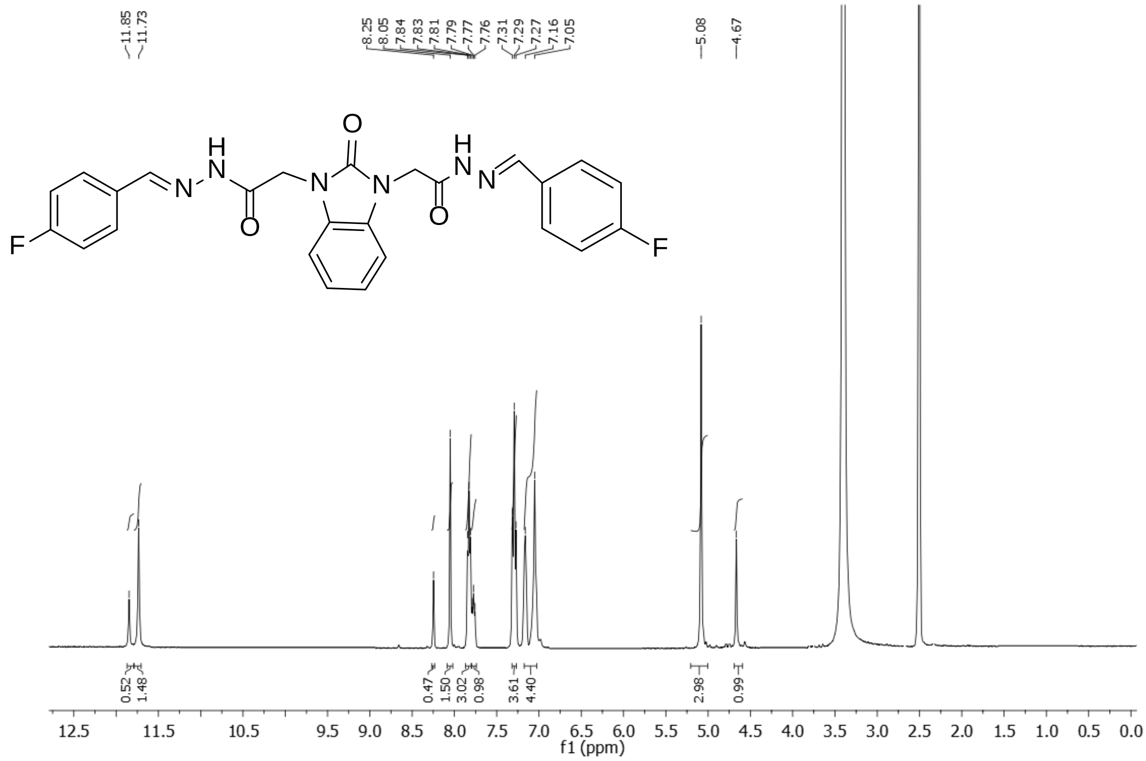

Figure S14. ^{1}HNMR of N,N-2,2'-(2-oxo-1*H*-benzo[*d*]imidazole-1,3(2*H*)-diyl)bis(4 flourobenzylidene)acetohydrazide).

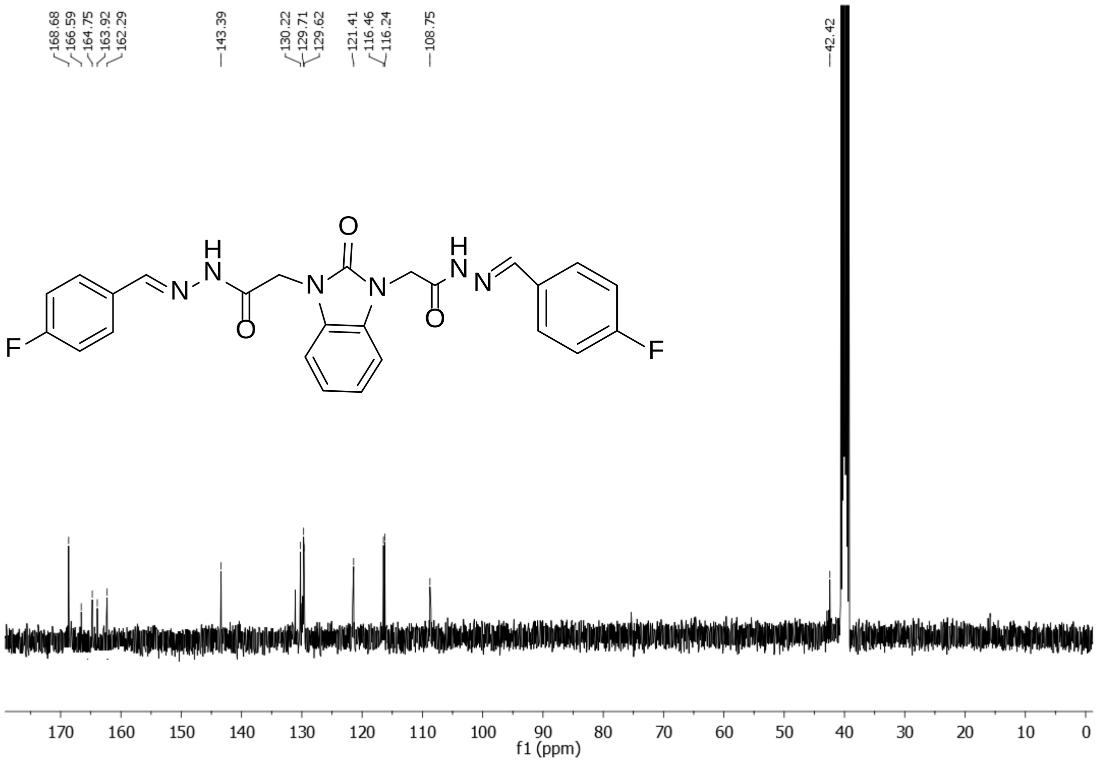

Figure S15. ^{13}CNMR of N,N-2,2'-(2-oxo-1*H*-benzo[*d*]imidazole-1,3(2*H*)-diyl)bis(4-flourobenzylidene)acetohydrazide).

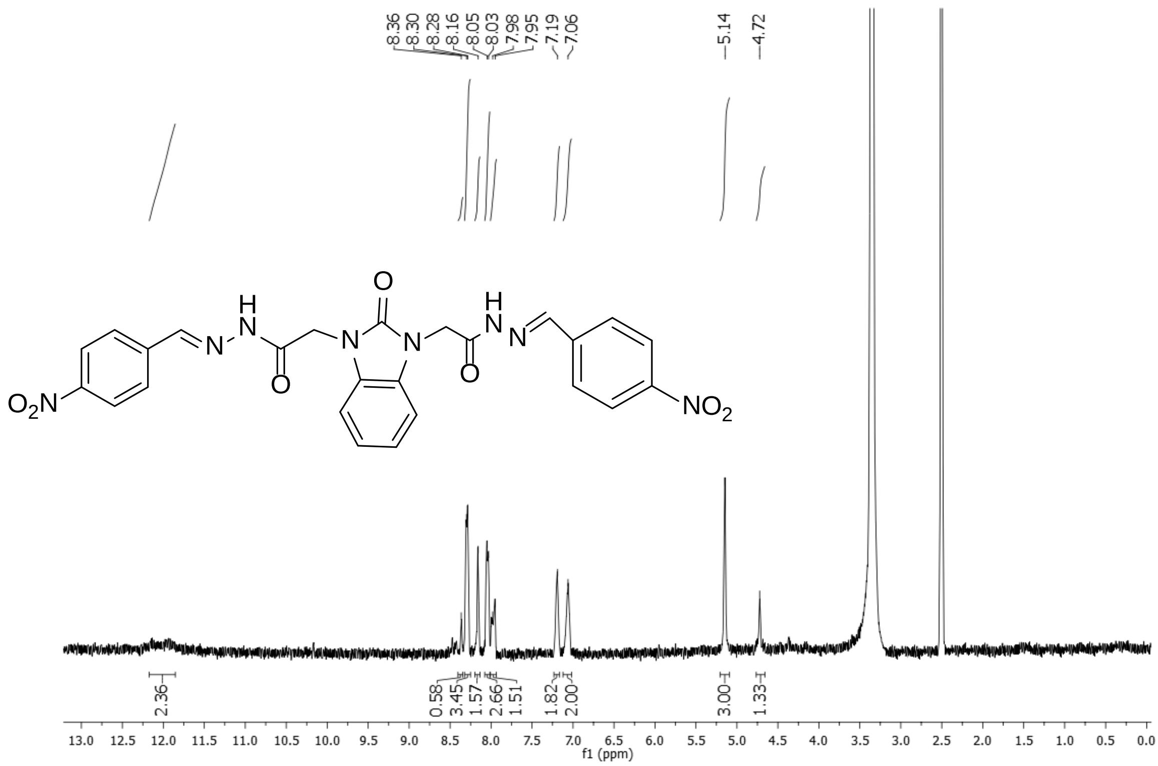

Figure S16. ^{1}HNMR of N,N-2,2'-(2-oxo-1*H*-benzo[*d*]imidazole-1,3(2*H*)-diyl)bis(4-nitrobenzylidene)acetohydrazide).
